# Iterative data-driven forecasting of the transmission and management of SARS-CoV-2/COVID-19 using social interventions at the county-level

**DOI:** 10.1038/s41598-022-04899-4

**Published:** 2022-01-18

**Authors:** Ken Newcomb, Morgan E. Smith, Rose E. Donohue, Sebastian Wyngaard, Caleb Reinking, Christopher R. Sweet, Marissa J. Levine, Thomas R. Unnasch, Edwin Michael

**Affiliations:** 1 Center for Global Health Infectious Disease Research, University of South Florida, Tampa, FL USA; 2 Department of Biological Sciences, University of Notre Dame, Notre Dame, IN USA; 3 Center for Research Computing, University of Notre Dame, Notre Dame, IN USA; 4 Center for Leadership in Public Health Practice, University of South Florida, Tampa, FL USA

**Keywords:** Diseases, Infectious diseases, Ecology, Ecological modelling, Population dynamics, Mathematics and computing, Applied mathematics, Computational science, Information technology, Scientific data

## Abstract

The control of the initial outbreak and spread of SARS-CoV-2/COVID-19 via the application of population-wide non-pharmaceutical mitigation measures have led to remarkable successes in dampening the pandemic globally. However, with countries beginning to ease or lift these measures fully to restart activities, concern is growing regarding the impacts that such reopening of societies could have on the subsequent transmission of the virus. While mathematical models of COVID-19 transmission have played important roles in evaluating the impacts of these measures for curbing virus transmission, a key need is for models that are able to effectively capture the effects of the spatial and social heterogeneities that drive the epidemic dynamics observed at the local community level. Iterative forecasting that uses new incoming epidemiological and social behavioral data to sequentially update locally-applicable transmission models can overcome this gap, potentially resulting in better predictions and policy actions. Here, we present the development of one such data-driven iterative modelling tool based on publicly available data and an extended SEIR model for forecasting SARS-CoV-2 at the county level in the United States. Using data from the state of Florida, we demonstrate the utility of such a system for exploring the outcomes of the social measures proposed by policy makers for containing the course of the pandemic. We provide comprehensive results showing how the locally identified models could be employed for accessing the impacts and societal tradeoffs of using specific social protective strategies. We conclude that it could have been possible to lift the more disruptive social interventions related to movement restriction/social distancing measures earlier if these were accompanied by widespread testing and contact tracing. These intensified social interventions could have potentially also brought about the control of the epidemic in low- and some medium-incidence county settings first, supporting the development and deployment of a geographically-phased approach to reopening the economy of Florida. We have made our data-driven forecasting system publicly available for policymakers and health officials to use in their own locales, so that a more efficient coordinated strategy for controlling SARS-CoV-2 region-wide can be developed and successfully implemented.

## Introduction

The implementation of population-wide non-pharmaceutical socially-based suppressive measures, focused on lockdowns of whole communities, social distancing, travel restrictions, and increasingly the deployment of testing and contact tracing^1–3^, has led to remarkable success in dampening the initial waves of the ongoing severe acute respiratory syndrome-coronavirus 2 (SARS-CoV-2/COVID-19) pandemic globally (https://aatishb.com/covidtrends/). This has resulted in many countries attempting to lift these unprecedented behavioral measures to allow the re-opening of their economies while awaiting the arrival and mass administration of less socially-disruptive technologies, such as viable vaccines^4^. Health officials are, however, becoming increasingly concerned about the likely impacts that such re-openings could have on the subsequent transmission dynamics of the virus. One reason for this concern is that in many areas, easing of the above non-pharmaceutical interventions (NPIs) has taken place before the initial epidemics have reached their endpoints. Another is that herd immunity in communities that have been under these interventions has not developed to levels that would mitigate the possibility of infection resurgences^5,6^. These possibilities, including the economic and political imperatives for easing NPIs, have led to increased attention being paid to the identification and deployment of those social measures that will enable the containment of viral transmission to levels that would allow reopening of societies with minimal harmful health and social side-effects^7–10^.

A distinctive feature of the policy response to the management of the COVID-19 pandemic worldwide has been the role played by epidemiological modelling for evaluating the use of behavioral interventions exclusively for controlling epidemic outbreaks in populations^8–15^ These mathematical models, based primarily on extensions to the standard SEIR epidemic model, but also newer methods based on machine learning, network analysis, agent-based simulations, and empirical growth models based on incidence data^16–19^, have enabled predictions of the course of the epidemic to warn policy-makers of the gravity of potential impacts, as well as help them in making comparisons of the various social measures proposed for suppressing viral transmission in exposed communities. For example, these tools have played critical roles for evaluating the comparative effects of locking down communities versus allowing a portion of the population to be exposed and develop immunity as alternate strategies for containing both first and subsequent epidemic waves^20,21^.

However, while these models have been important for supporting generalized, scenario-based, policy planning, they can be less useful for simulating dynamics after the disease breaks out in real communities^19^. First, such models have little support for capturing the rapidly changing social contexts in which an epidemic evolves. This is an important need since for fast evolving phenomena, such as an epidemic, feedbacks from changing conditions need to be processed speedily to correct for the invariable modelling errors surrounding a dynamic forecast^19^. Second, addressing forecasts of the near future as an epidemic develops requires models that give accurate forecasts with the least variance at the lead times required by management^22^. Third, general models, even if supported by the available historical data, do not address the need for data assimilation in real-time forecasting. Such data assimilation, in which information regarding the extant transmission processes that are embedded in observational data is used to iteratively update the underlying dynamical principles represented by the structure and parameters of a model, has been shown to provide near-term forecasts of the state of a dynamical system which are better than could be obtained with just data or the model alone^23–28^. For forecasting epidemics over the near future, this data-model assimilation framework will allow shrinkage of forecast variance while also correcting for model bias and drift^29^. Finally, few currently available mathematical models of SARS-CoV-2 transmission provide predictions at lower administrative levels, such as the county^30,31^. Thus, they do not capture the spatial and social heterogeneity^32,33^ that drives epidemics in the real-world, and lack the resolution for evaluating interventions that take a fuller account of these heterogeneities at the lower spatial scales of an epidemic^19^.

Developing, testing and refining data-driven locally-applicable models for enabling reliable near- and long-term forecasts for decision-making can be a challenge especially when the objective is to control an ongoing epidemic^15^. One problem is the requirement that locally relevant observations are transmitted sufficiently rapidly to be useful for updating models within the lead times connected with making an effective public response. Fortunately, advances in communication systems, powerful data processing, storage software and hardware, and an increasing focus on the provision of standardized, open-source data for diseases, are now becoming available that are providing solutions to this problem^34–36^. In parallel, major progress has also been made in the development of iterative statistical data-model assimilation techniques, whereby data of diverse types and prior information regarding model structures and parameters can be used reliably to constrain model parameters or states in a setting, including supporting evaluations of forecast uncertainty over time^22,23,25,37–44^. Lastly, developments in cyberinfrastructures to automate the dynamic integration of new data and information to facilitate regular assessment of forecasts and active updating of models mean that the practical implementation of iterative data-driven locally-applicable epidemic forecasting is now increasingly becoming possible^27,36,38,39,45–53^.

This paper describes the efforts of our team to develop and use such an iterative data-model assimilation-based forecasting system for SARS-CoV-2, wherein we use a SEIR-type model updated sequentially with publicly available COVID-19 case and human movement data in order to provide predictions of the course of the pandemic under various social interventions at the county level in the United States. Here, we report on the use of these data from counties in the state of Florida to demonstrate how the developed data-driven modelling system can allow forecasts of the localized epidemic dynamics as well as enable evaluations of the relative impacts of the social interventions until mass vaccination strategies can come into play in the state. We also examine how gaining a better understanding of the geographical variation in the propagation of the virus and our attempts to curtail it can allow the derivation of a regionally-varying or tailored response that is effective at minimizing contagion whilst offering at the same time the advantages of less restrictive rules for parts of a state’s population.

## Results

### Model fits to data, validation, and assessment of geographic drivers of transmission

**Figure 1:**
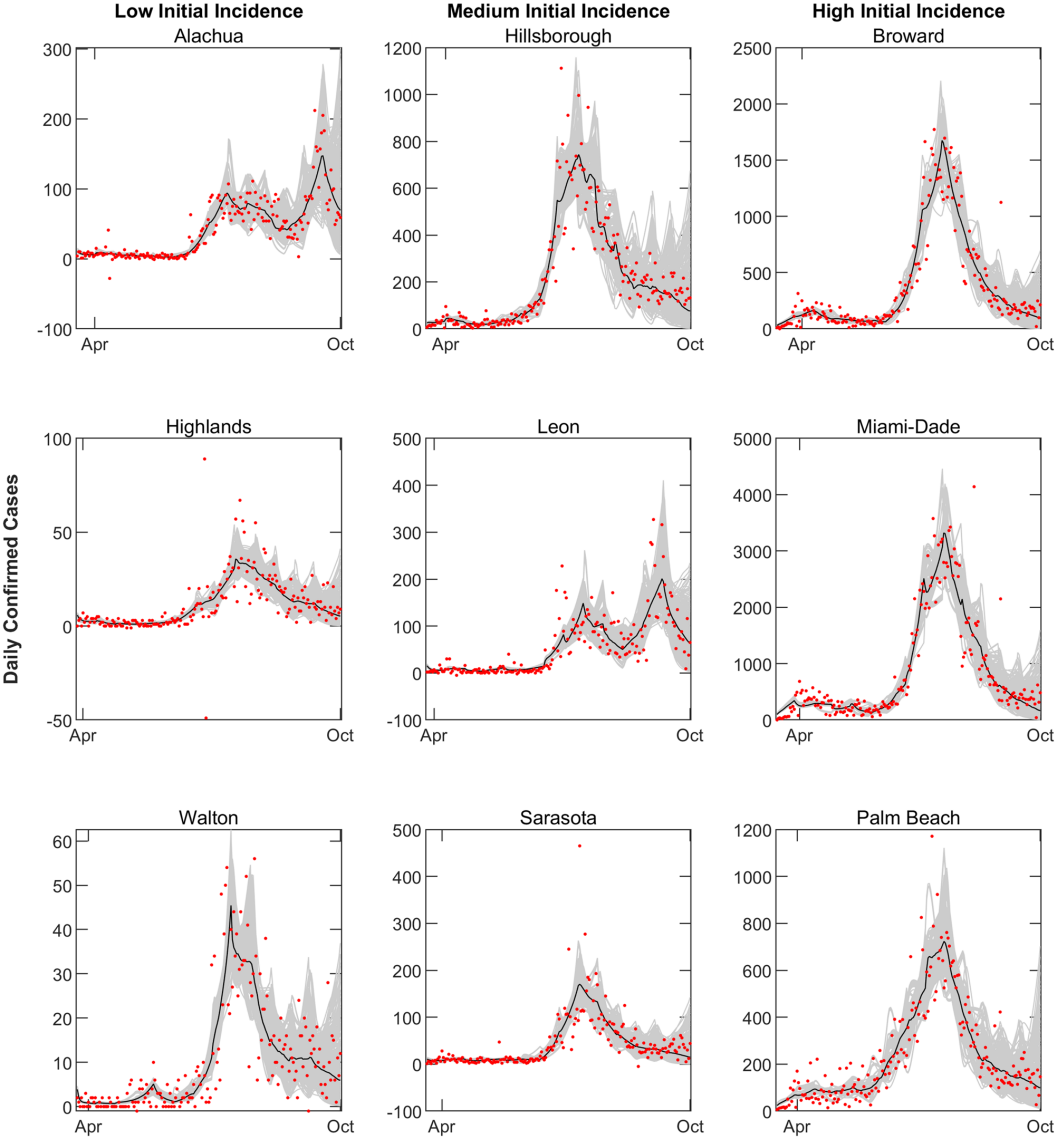
Model fits compared to confirmed daily case data in 9 representative Florida counties. Gray curves represent county-specific model predictions and red points represent confirmed case data obtained from Johns Hopkins University^47^. Fitting was started after at least 10 confirmed cases were reported. Results from model fits to representative countries, stratified by initial incidence growth rate in each county (Group a < 0.05, Group b > 0.05–0.15, Group c > 0.15) are shown. Group a counties: Alachua, Highlands, and Walton; group b: Hillsborough, Leon, and Sarasota; and group c: Broward, Miami-Dade, and Palm Beach.

We modeled the observed SARS-CoV-2 outbreaks in the 67 counties of Florida (Supplementary Table S1) based on reported case and mortality data, and information regarding local social behaviors, through to September 30th, 2020. This was carried out by using a Bayesian melding data assimilation approach centered on the sequential calibration of our compartmental epidemic model to confirmed case and death time series data, and data on human movements, reported for each of these counties by the Johns Hopkins University Coronavirus Resource Center^47^ and the location data firm, Unacast (https://www.unacast.com/covid19/social-distancing-scoreboard), respectively (see “Methods”). The model predictions for daily confirmed cases compared to data for nine representative Florida counties are shown in Fig. 1. The results show that the 95% confidence interval bounds of the predictions from the ensemble of sequentially updated models for each county are able to envelop nearly all of the confirmed case data over the calibration period. The corresponding model fits to cumulative confirmed cases from all Florida counties are portrayed in Supplementary Fig. S1.

Because the dynamics of COVID-19 are significantly influenced by changes in social behaviors, it is critical to iteratively calibrate transmission models, such as the present SEIR model, to new data and actively update the resulting predictions accordingly^24,27^. Supplementary Fig. S2 demonstrates the shift in model predictive performance as the model is updated sequentially with various lengths of incoming longitudinal data. Cross-validation analysis showed that carrying out such model calibrations to 2-week sequential blocks of data can maintain the relative mean-square error to consistently below 20%, while also being computationally feasible. Implementing this 2-week sequential updating procedure thus allowed us to incorporate information regarding changing transmission conditions in each county as effectively as possible into the model.

Supplementary Table S2 summarizes the values of the posteriors obtained by model fitting to the 14-day case day prior to September 30th for the social distancing parameter, d, which modifies the transmission rate, β, in each county, as well as for the fraction of the respective county population remaining under mobility restriction as estimated using the Unacast mobility data (see “Methods”).The results show that while the values of each of these two key social parameters varied between the present counties, the values of the social distancing parameter, d, appears to be comparatively less variable compared to those estimated for the lockdown fractions (Supplementary Table S2). This suggests that the between-county variations in SARS-CoV-2 outbreak dynamics, and the individual county-level response to the intervention scenarios, reported here (from October 1st 2020) may be a reflection of the combined effect of the initial incidences and variations in the numbers of the susceptible populations in a county that are released from restricted movement.

### Forecasting the epidemic and impacts of social interventions

**Figure 2:**
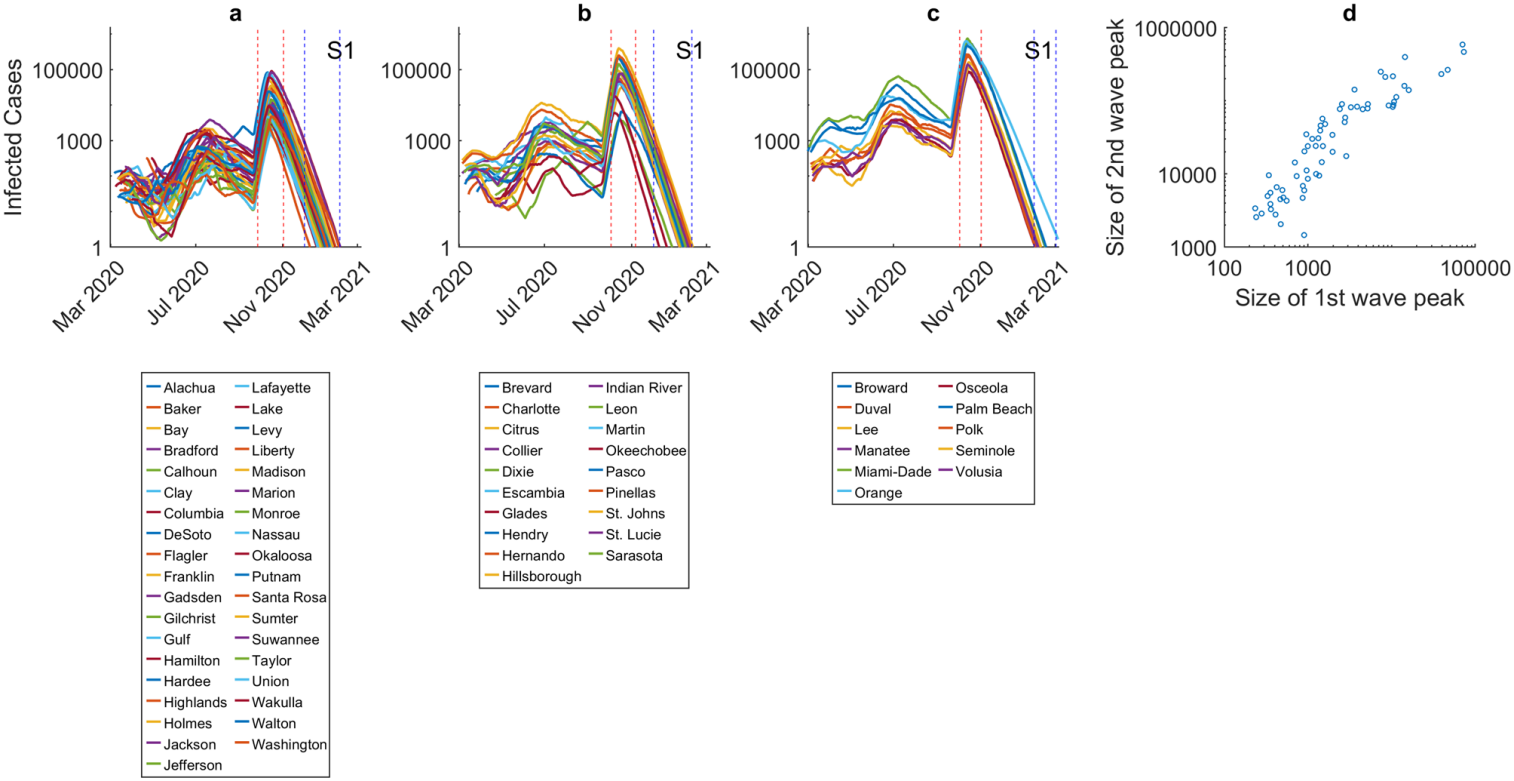
Epidemic forecasts for individual counties in the scenario where all interventions are lifted following the initial lockdown (S1). Each curve represents the median prediction for a given county. The results are stratified by initial incidence growth rate in each county (Group a < 0.05, Group b > 0.05– 0.15, Group c > 0.15). The intervention scenario (also see Supplementary Fig. S3) represents a full release of lockdown and social distancing from October 1st. The y-axis is shown in log-scale to better visualize the difference between the size of the first and second epidemic waves. The range of epidemic peaks and epidemic ending dates for each group is shown by red and blue dotted vertical lines, respectively. The 2nd wave peaks of infected cases occurs between September 27th–November 2nd for Group a, October 3rd–November 7th for group b, and October 3rd–November 2nd for Group c. The epidemic ending dates occur between December 2nd–January 21st, 2021 for Group a, December 2nd–January 25th, 2021 for group b, and January 16th, 2021–March 2nd, 2021 for Group c, respectively.

**Figure 3:**
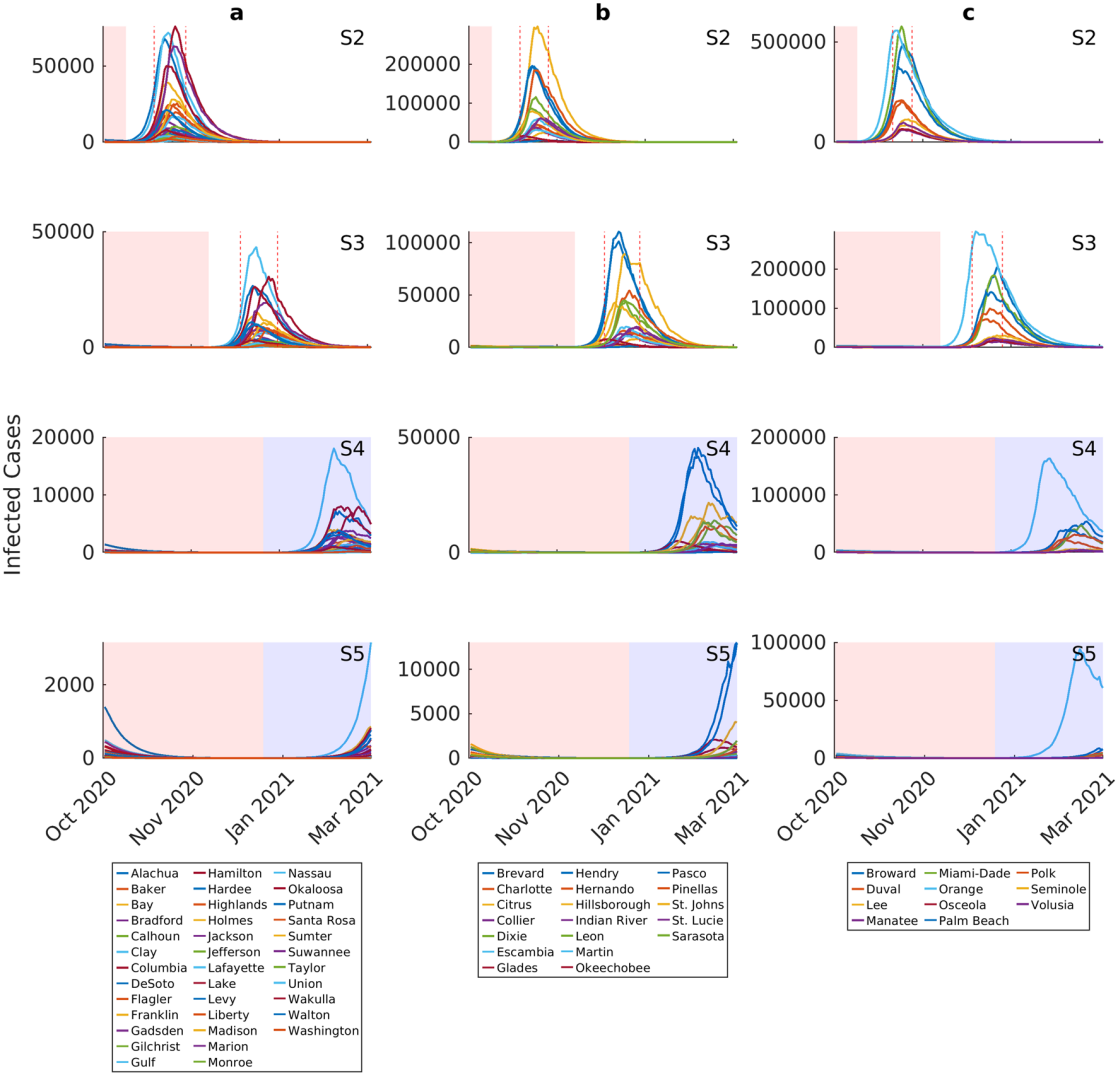
Epidemic forecasts for individual counties under four different social intervention scenarios. Note the differences in y-axis values when comparing scenarios. Each curve represents the median prediction for a given county. The results are stratified by initial incidence growth rates observed in each county (Group b < 0.05, Group b > 0.05–0.15, Group c > 0.15). The intervention scenarios are as follows (also see Supplementary Fig. S2): S2: maintain movement estimate and social distancing measures over 2 weeks from October 1st to October 14th; S3: maintain movement estimate and social distancing measures over 8 weeks from October 1st to November 30th; S4: maintain movement estimate and social distancing measures through end December 2020 + begin low intensity contact tracing and quarantine efforts on October 1st through March 2021; S5: maintain movement estimate and social distancing measures through end December 2020 + begin high intensity contact tracing and quarantine efforts on October 1st through March 2021. The red shading in each plot indicates the duration of movement and social distancing in each scenario, while the blue shading shows quarantining measures without movement restrictions or social distancing. The range of 2nd wave infection peaks for each county group is shown by dotted vertical lines. For scenario 2, the peak of infected cases occurs between October 29th–November 16th for Group a, October 29th–November 14th for group b, and November 2nd–November 13th for Group c. For scenario 3, the peak of infected cases occurs between December 17th–January 7th 2021 for Group a, December 16th–January 5th 2021 for group b, and December 17th–January 3rd 2021 for Group c. Epidemic ending dates for scenario 2 occur between January 2nd–February 17th 2021 for Group a, January 6th–February 21th 2021 for group b, and February 11th–February 27th, 2021 for Group c, respectively. For scenario 3, the corresponding end dates were between February 20th–March 2nd 2021 for Group a, and between February 22nd to beyond March 2nd for Groups b and c.

We used the models updated using the infection/death data reported to September 30th 2020 in each county to simulate both the local epidemic dynamics and compare the dynamical impacts of six different social interventions as described in “Methods” and depicted in Supplementary Fig. S3. The model predictions for infected cases under each intervention scenario through the end of the year are shown in Figs. 2 and 3, stratified by initial incidence growth rate (Group a < 0.05, Group b > 0.05–0.15, Group c > 0.15; see “Methods”). Scenario 1 is the least aggressive option considered with no interventions put in place and a full release of strict stay-at-home orders after September 30th. The results for this scenario show that because the social restrictions in Florida were lifted before the first epidemics ended, resurgence of the epidemic will be inevitable in every county (Fig. 2). Indeed, an average of 27% (with a range of 15–47%) of the overall population across all counties is projected to become infected at the peaks of the resulting 2nd waves under this scenario (Fig. 2, Table 1, Supplementary Table S1). Note that these, and subsequent model forecasts, account for all infected cases, including those who are not yet infectious (exposed class), asymptomatic, presymptomatic, and symptomatic. The county level model predictions from this scenario also highlight the variation in the peak size of the 2nd waves that different counties could have faced with the lifting of all interventions, with these sizes ranging from 1459 cases in Lafayette to as high as 742,898 cases in Miami-Dade County (Table 1). The time course of the epidemic is also variable with higher incidence counties expected to see generally later peaks compared to lower incidence counties (Fig. 2). Figure 2d shows that the predicted size of the 2nd wave peaks are directly related to those of the first waves that occurred in each county, further underlining the impact that initial variation in local conditions of virus transmission can have on the size of subsequent county-level infection resurgences in each county. The simulations also reveal that while the size of the 2nd waves will be large with the full release of lockdowns, the epidemics in each county will nonetheless, as expected, eventually end (Fig. 2), with the possibility that this will occur earlier in the case of the low incidence counties compared to the case with high incidence counties where the corresponding 2nd waves are predicted to end much later.

**Table 1:** Total median infected cases predicted at the epidemic peak in each county. The 90% confidence interval is given in brackets. Counties are stratified by initial county-level incidence growth rate.

Class	County	Peak total infected cases								
		S1			S2		S3	S4		S5
Group A	Alachua	82,696 [1070–1596,94]			67,899 [437–153,417]		26,572 [437–137,973]	7161 [0–114,585]		767 [0–86,530]
	Baker	6856 [99–16,785]			5121 [42–15,844]		1769 [42–12,989]	696 [0–10,974]		70 [0–8338]
	Bay	51,096 [453–104,718]			39,280 [153–101,090]		14,864 [83–91,565]	3991 [0–73,713]		862 [0–61,507]
	Bradford	9479 [564–15,862]			9000 [255–15,293]		5917 [52–13,820]	2557 [0–11,390]		342 [0–8596]
	Calhoun	2567 [4–7667]			1830 [1–7505]		496 [1–7008]	89 [0–5805]		1 [0–4177]
	Clay	78,651 [2997–130,822]			71,980 [1190–129,958]		43,312 [112–124,650]	18,053 [0–103,965]		3157 [0–78,202]
	Columbia	20,045 [187–39,534]			17,378 [63–38,397]		9488 [24–34,992]	3496 [0–29,300]		510 [0–22,444]
	DeSoto	6013 [20–21,142]			4831 [6–20,810]		1582 [2–19,252]	348 [0–15,799]		7 [0–11,807]
	Flagler	23,992 [151–69,097]			19,676 [40–68,831]		7942 [40–62,499]	1571 [0–52,534]		44 [0–41,203]
	Franklin	2875 [7–6756]			2234 [2–6554]		755 [2–5851]	211 [0–4886]		39 [0–3715]
	Gadsden	11,163 [101–23,173]			9126 [33–22,193]		3767 [33–20,450]	1268 [0–16,804]		66 [0–11,840]
	Gilchrist	3386 [17–10,791]			2630 [5–10,694]		871 [3–9089]	197 [0–7233]		8 [0–6079]
	Gulf	2057 [4–6804]			1483 [1–6659]		375 [1–6284]	61 [0–5050]		1 [0–3730]
	Hamilton	4298 [52–7749]			3550 [13–7593]		1479 [11–6955]	577 [0–5884]		135 [0–4657]
	Hardee	8587 [182–15,067]			6977 [62–14,628]		3053 [21–12,988]	1274 [0–11,027]		205 [0–8255]
	Highlands	30,159 [151–65,762]			24,480 [36–64,131]		8742 [22–58,161]	2160 [0–48,123]		257 [0–38,953]
	Holmes	5527 [35–11,360]			4437 [11–11,072]		1649 [8–9886]	551 [0–8216]		86 [0–6447]
	Jackson	14,501 [742–24,908]			13,119 [323–24,227]		7631 [39–22,824]	2737 [0–19,021]		236 [0–14,161]
	Jefferson	3279 [15–7879]			2608 [5–7648]		968 [4–6670]	454 [0–5540]		75 [0–4352]
	Lafayette	1459 [8–3692]			1014 [3–3526]		275 [3–3196]	50 [0–2624]		0 [0–1851]
	Lake	90,197 [672–219,255]			76,121 [225–217,105]		30,544 [58–204,774]	7937 [0–168,674]		225 [0–123,828]
	Levy	9600 [34–24,021]			8061 [11–23,800]		3621 [6–21,247]	1002 [0–17,559]		70 [0–13,410]
	Liberty	2782 [74–4093]			2502 [29–3959]		1522 [3–3521]	809 [0–2923]		319 [0–2352]
	Madison	4990 [31–10,049]			4266 [9–9950]		1878 [6–9422]	540 [0–7749]		21 [0–5907]
	Marion	83,145 [715–210,699]			63,118 [191–206,400]		19,313 [83–190,635]	3779 [0–158,420]		159 [0–118,195]
	Monroe	14,350 [21–43,786]			10,199 [5–43,207]		2954 [4–39,815]	633 [0–32,372]		31 [0–25,890]
	Nassau	20,362 [134–51,309]			16,705 [37–50,465]		6450 [37–45,458]	1412 [0–38,193]		55 [0–28,346]
	Okaloosa	58,920 [805–127,741]			50,121 [261–124,682]		26,221 [40–118,424]	8021 [0–99,617]		820 [0–75,945]
	Putnam	24,005 [310–44,369]			21,297 [99–43,149]		10,829 [33–39,626]	3847 [0–33,244]		549 [0–25,607]
	Santa Rosa	34,202 [91–108,943]			25,531 [27–106,705]		7368 [27–101,151]	1225 [0–84,021]		19 [0–63,872]
	Sumter	34,849 [109–81,727]			28,042 [26–80,734]		10,592 [19–75,813]	2447 [0–64,093]		87 [0–50,568]
	Suwannee	9989 [60–24,326]			7334 [16–24,050]		2051 [16–21,234]	430 [0–17,613]		29 [0–14,022]
	Taylor	4511 [11–11,219]			3477 [3–10,838]		1179 [3–9750]	398 [0–7979]		23 [0–5901]
	Union	4701 [274–7989]			3926 [85–7800]		1886 [50–7089]	561 [0–5789]		24 [0–4476]
	Wakulla	9333 [53–19,843]			7997 [23–19,083]		3061 [23–17,562]	975 [0–14,366]		83 [0–10,707]
	Walton	24,138 [192–44,473]			21,068 [57–43,626]		10,072 [19–40,425]	3227 [0–34,005]		638 [0–27,300]
	Washington	4756 [7–14,187]			3660 [2–13901]		1135 [2–12,619]	207 [0–10,546]		5 [0–7753]
Group B	Brevard	216,546 [5203–382,548]			194,327 [2387–377,392]		101,280 [206–335,789]	41,631 [1–289,509]		13,001 [0–230,793]
	Charlotte	56,932 [203–120,293]			44,307 [50–118,087]		15,841 [25–110,865]	4173 [0–93,810]		814 [0–72,942]
	Citrus	30,776 [334–90,613]			24,683 [89–90,663]		7968 [46–84,041]	1431 [0–71,002]		23 [0–54,232]
	Collier	81,605 [217–222,097]			61,625 [54–218,579]		19,574 [54–208,409]	3953 [0–177,533]		117 [0–134,752]
	Dixie	3875 [14–9253]			3071 [3–9105]		1189 [3–8365]	235 [0–7096]		6 [0–5327]
	Escambia	76,781 [225–185,633]			59,268 [55–180,840]		19,828 [39–164,626]	4584 [0–135,057]		449 [0–106,358]
	Glades	5965 [1024–8090]			5406 [659–7903]		3615 [229–7062]	2348 [34–5938]		1176 [0–4848]
	Hendry	6591 [26–22,832]			5053 [6–23,017]		1469 [3–20,760]	268 [0–17,420]		7 [0–13,207]
	Hernando	48,155 [323–116,496]			38,298 [86–114,877]		13,378 [42–105,215]	3046 [0–86,793]		120 [0–69,350]
	Hillsborough	397,091 [1516–871,468]			296,947 [354–850,775]		89,133 [353–791,987]	21,603 [0–658,441]		1619 [0–529,576]
	Indian River	45,901 [301–99,331]			37,314 [94–98,143]		12,981 [16–91,537]	3739 [0–79,991]		721 [0–61,596]
	Leon	96,958 [3129–170,833]			85,935 [1106–169,586]		43,604 [362–154,062]	13,896 [0–131,224]		513 [0–101,078]
	Martin	39,199 [148–94,308]			31,207 [40–91,940]		10,958 [26–84,112]	2816 [0–72,332]		280 [0–55,257]
	Okeechobee	17,530 [587–25,182]			15,780 [205–24,052]		7888 [58–20,736]	4710 [0–17,210]		2084 [0–14,028]
	Pasco	211,838 [5739–341,641]			195,573 [2836–338,437]		110,606 [146–313,045]	45,360 [1–262,734]		13,023 [0–217,987]
	Pinellas	249,728 [1126–594,592]			184,207 [361–580,025]		54,312 [123–533,053]	11,694 [0–433,138]		1090 [0–344,081]
	St. Johns	90,187 [1960–164,989]			79,325 [711–162,425]		42,894 [71–147,866]	15,740 [0–123,935]		4092 [0–98,928]
	St. Lucie	77,214 [335–197,173]			61,019 [97–194,291]		18,514 [65–178,646]	3568 [0–146,898]		115 [0–110,119]
	Sarasota	142,268 [656–277,025]			115,675 [193–269,067]		44,632 [41–252,870]	13,217 [0–217,234]		1961 [0–170,587]
Group C	Broward	587,916 [4074–1,242,206]			488,812 [1248–1,227,896]		204,061 [394–1,156,885]	53,764 [0–1,006,656]		7621 [0–791,455]
	Duval	265,303 [1948–570,314]			208,691 [598–553,209]		71,687 [309–501,103]	22,480 [0–415,914]		4270 [0–332,061]
	Lee	160,090 [586–455,057]			113,444 [109–453,325]		30,393 [109–421,305]	4979 [0–358,649]		287 [0–285,072]
	Manatee	86,346 [296–245,053]			60,866 [89–238,941]		15,092 [89–209,427]	2743 [0–174,291]		195 [0–144,646]
	Miami-Dade	742,898 [3860–1,564,957]			577,662 [1066–1,553,004]		183,730 [621–1,386,649]	47,087 [0–1,157,549]		3782 [0–948,964]
	Orange	625,542 [52885–988,098]			557,470 [30133–973,832]		295,526 [2752–870,235]	163,365 [29–767,373]		94,256 [1–687,917]
	Osceola	87,801 [200–221,442]			64,189 [62–216,093]		18,324 [62–193,890]	3667 [0–165,232]		227 [0–122,945]
	Palm Beach	466,637 [8530–990,986]			376,919 [2934–973,697]		141,320 [555–900,048]	39,597 [0–789,726]		8471 [0–633,199]
	Polk	232,456 [3279–434,136]			203,981 [1353–421,046]		99,111 [180–398,978]	31,266 [0–333,647]		3092 [0–255,211]
	Seminole	112,562 [215–282,767]			85,203 [75–278,362]		25,402 [75–253,855]	5711 [0–210,925]		696 [0–173,157]
	Volusia	140,178 [1310–343,332]			97,138 [383–336,873]		23,400 [152–309,346]	4716 [0–265,955]		380 [0–212,424]
Total		6,206,385			5,035,473		2,009,893	662,339		174,513

The inclusion of social distancing measures through October and November in scenarios 2 and 3, respectively, is predicted to have two key effects (Fig. 3). First, it is to be noted that these measures will not prevent the occurrence of sizable 2nd waves; however, they will shift the timing of the county-level 2nd epidemic peaks further into the future, with this shift more pronounced for scenario 3 (from the October/November peaks predicted for scenario 2 to the December/January peaks forecasted for scenario 3; see legend to Fig. 3). These measures, however, will significantly reduce the size of the 2nd epidemic peaks, with the more intensive scenario 3 bringing about an average case reduction of 76% (with a range of 37–83%) compared to scenario 1 (Table 1). Again, these outcomes will vary by county group, with shifts to 2nd peaks, and resolution of the epidemics, occurring generally later among the high incidence countries (Group c), while the greatest reduction in peak cases will occur for the low incidence counties (Fig. 3, Table 1).

Implementation of contact tracing and quarantine measures to prevent a fraction of the infectious population from spreading the disease (25% quarantine rate in scenario 4 and 50% quarantine rate in scenario 5 both through March 2021) along with sustained social distancing measures through December 2020, is predicted to have a uniformly high suppressive effect on the course of the 2nd waves in each county. Scenario 4 reduces the size of the averaged county-level epidemic peak to affect just 3% of the total population (with a range of 0–17%), while scenario 5 is predicted to reduce the average peak infection size to just 0.6% of the overall population in the state (with a range of 0–8.5%). This represents an average peak reduction compared to scenario 1 of 89% (with a range of 61–97%) for scenario 4 and 98% (with a range of 80–100%) for scenario 5. Furthermore, the results show that scenario 5 could even bring about breakage of epidemic transmission in some counties, particularly in the case of those that exhibited the lowest initial infection incidences (Table 1). Interestingly, these intervention scenarios also appear to generally lessen the between-county group variations in the timings and peaks of the predicted 2nd epidemics (Fig. 3).

**Figure 4:**
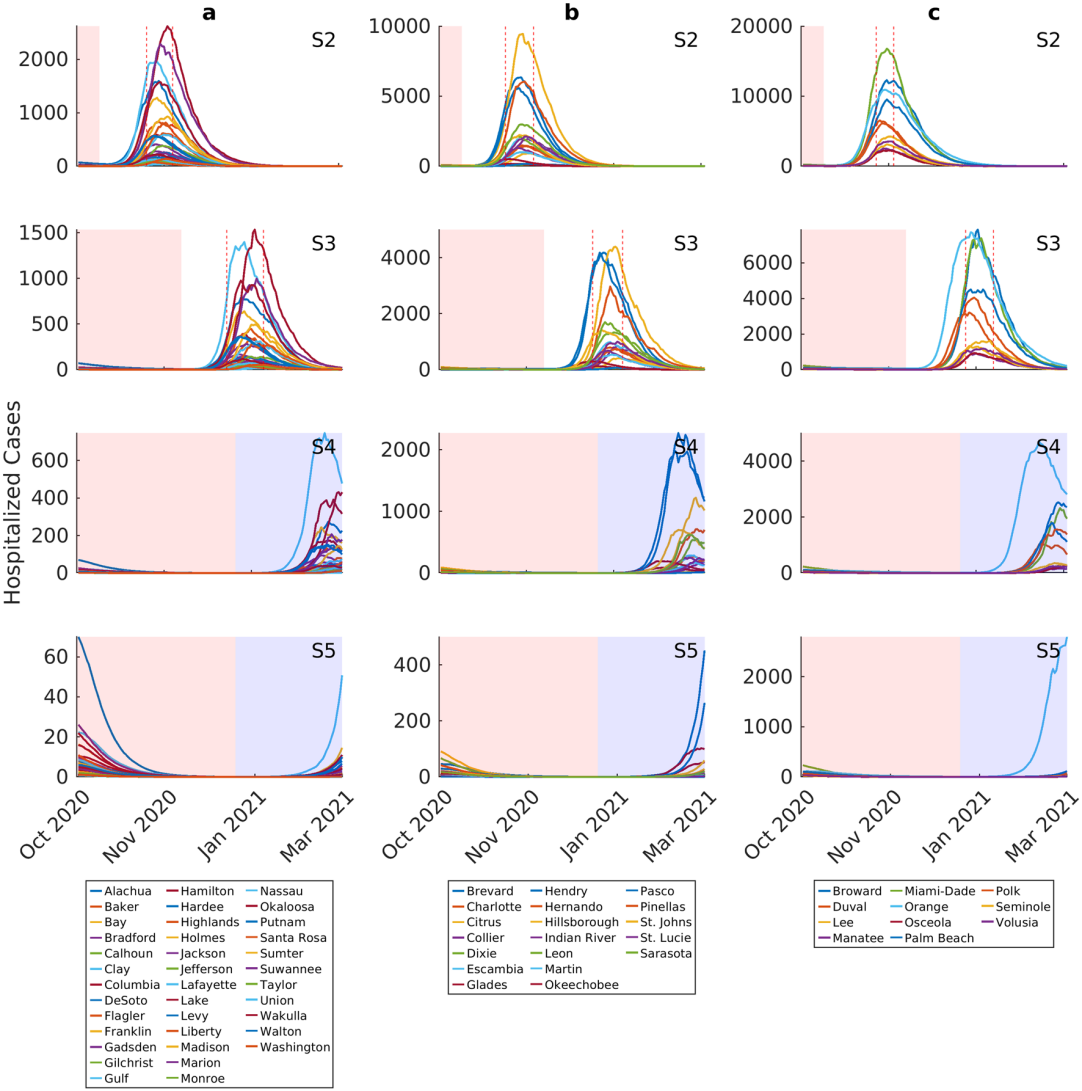
Forecasts of corresponding hospitalized cases for individual counties under the four different social intervention scenarios. Hospitalized cases include both hospital and ICU model compartments. Note the differences in y-axis values when comparing scenarios. Each curve represents the median prediction for a given county. The results are stratified by initial county-level incidence growth rate (Group a < 0.05, Group b > 0.05–0.15, Group c > 0.15). The intervention scenarios are as follows (also see Supplementary Fig. S2): S2: maintain movement estimate and social distancing measures over 2 weeks from October 1st to October 14th; S3: maintain movement estimate and social distancing measures over 8 weeks from October 1st to November 30th; S4: maintain movement estimate and social distancing measures through end December 2020 + begin low intensity contact tracing and quarantine efforts on October 1st through March 2021; S5: maintain movement estimate and social distancing measures through end December 2020 + begin high intensity contact tracing and quarantine efforts on October 1st through March 2021. The red shading in each plot indicates the duration of movement and social distancing in each scenario, while the blue shading shows quarantining measures without movement or social distancing. The range of peak hospitalized cases for each group is shown by dotted vertical lines. For scenario 2, the peak of hospitalized cases occurs between November 9th–November 24th for Group a, November 7th–November 23rd for group b, and November 12th–November 22nd for Group c. For scenario 3, the peak of hospitalized cases occurs between December 25th–January 15th 2021 for Group a, December 27nd–January 13th 2021 for group b, and January 2nd, 2021–January 18th 2021 for Group c.

The above scenario differences are also apparent in the predictions of the required hospitalizations at the county level (Fig. 4). However, the hospitalization forecasts also indicate that without the more aggressive interventions, such as those modeled in scenarios 4 and 5, there would be a high risk that predicted cases will exceed existing county-level hospital capacities (Table 2). This serious outcome will also vary significantly by geographic location.

**Table 2:** Total hospitalized cases predicted at the epidemic peak in each county. The 90% confidence interval is given in brackets. Bold cells denote situations when hospitalization cases are predicted to be below the corresponding county-level care (bed/ICU) capacity. Counties are stratified by initial county-level incidence growth rate.

Class	County	Peak hospitalized cases					Bed capacity
		S1	S2	S3	S4	S5	
Group A	Alachua	1966 [40–8885]	1596 [20–8678]	791 [20–7262]	**274 [0–6270]**	**8 [0–4574]**	739
	Baker	201 [4–931]	167 [2–850]	65 [2–624]	25 [0–491]	**1 [0–435]**	17
	Bay	1504 [20–6190]	1281 [5–5930]	640 [5–5222]	244 [0–4351]	**15 [0–3551]**	125
	Bradford	230 [18–829]	217 [9–803]	166 [2–689]	95 [0–548]	**6 [0–426]**	17
	Calhoun	75 [0–440]	59 [0–406]	24 [0–347]	**4 [0–273]**	**0 [0–194]**	5
	Clay	2095 [99–7779]	1955 [38–7690]	1398 [5–6750]	745 [0–5660]	**51 [0–4560]**	138
	Columbia	635 [8–2491]	588 [3–2436]	389 [2–2145]	173 [0–1822]	**10 [0–1389]**	147
	DeSoto	224 [1–1282]	183 [0–1251]	82 [0–1046]	18 [0–883]	**0 [0–617]**	0
	Flagler	720 [6–3655]	614 [2–3580]	345 [2–2894]	80 [0–2374]	**1 [0–1930]**	43
	Franklin	85 [0–429]	70 [0–404]	34 [0–341]	**11 [0–282]**	**1 [0–221]**	15
	Gadsden	316 [4–1268]	277 [2–1222]	140 [2–1066]	57 [0–810]	**2 [0–617]**	0
	Gilchrist	97 [1–551]	80 [0–536]	34 [0–468]	9 [0–366]	**0 [0–259]**	0
	Gulf	75 [0–371]	59 [0–353]	22 [0–295]	**3 [0–251]**	**0 [0–163]**	13
	Hamilton	123 [1–557]	110 [1–549]	62 [1–458]	29 [0–389]	**2 [0–312]**	0
	Hardee	199 [7–871]	172 [2–832]	93 [1–645]	43 [0–548]	**4 [0–465]**	3
	Highlands	960 [6–4109]	827 [2–3990]	446 [2–3357]	137 [0–2665]	**5 [0–2196]**	121
	Holmes	149 [1–593]	128 [1–569]	61 [1–495]	21 [0–388]	**1 [0–322]**	9
	Jackson	444 [24–1580]	410 [11–1550]	281 [2–1394]	135 [0–1151]	**4 [0–834]**	56
	Jefferson	102 [0–465]	89 [0–455]	42 [0–377]	17 [0–301]	**2 [0–230]**	0
	Lafayette	47 [0–223]	37 [0–216]	14 [0–179]	2 [0–139]	**0 [0–98]**	0
	Lake	3017 [34–13,632]	2621 [11–13,489]	1534 [6–12,044]	431 [0–9821]	**4 [0–6676]**	287
	Levy	271 [2–1220]	246 [0–1218]	138 [0–1114]	46 [0–874]	**1 [0–644]**	0
	Liberty	79 [3–288]	73 [2–279]	48 [0–220]	32 [0–197]	**11 [0–167]**	0
	Madison	135 [1–615]	116 [0–589]	75 [0–548]	26 [0–464]	**0 [0–344]**	2
	Marion	2744 [23–12,978]	2286 [7–12,699]	997 [7–11,384]	**207 [0–9336]**	**3 [0–6933]**	455
	Monroe	474 [1–2576]	383 [0–2500]	143 [0–2178]	**33 [0–1731]**	**0 [0–1323]**	58
	Nassau	683 [5–3054]	591 [3–3020]	313 [3–2796]	74 [0–2307]	**1 [0–1667]**	60
	Okaloosa	1761 [24–7765]	1578 [9–7501]	976 [3–6601]	391 [0–5551]	**11 [0–4319]**	201
	Putnam	617 [11–2260]	553 [3–2235]	361 [2–2010]	151 [0–1610]	**7 [0–1325]**	36
	Santa Rosa	997 [3–5986]	810 [2–5780]	285 [2–5542]	**57 [0–4443]**	**0 [0–3263]**	176
	Sumter	1066 [4–5059]	931 [2–4997]	494 [2–4592]	143 [0–4080]	**2 [0–3219]**	44
	Suwannee	298 [2–1426]	240 [1–1380]	94 [1–1205]	20 [0–945]	**0 [0–725]**	13
	Taylor	136 [1–635]	114 [0–608]	50 [0–502]	**19 [0–410]**	**0 [0–326]**	20
	Union	151 [8–497]	126 [3–479]	73 [2–421]	**27 [0–334]**	**0 [0–255]**	33
	Wakulla	255 [2–1015]	216 [1–981]	120 [1–841]	39 [0–706]	**1 [0–535]**	0
	Walton	628 [5–2532]	570 [1–2429]	356 [1–2131]	145 [0–1767]	**10 [0–1234]**	130
	Washington	160 [0–822]	136 [0–803]	53 [0–703]	**11 [0–544]**	**0 [0–354]**	14
Group B	Brevard	6929 [254–23,234]	6357 [129–22,450]	4175 [10–19,815]	1980 [0–16,626]	**452 [0–140,52]**	756
	Charlotte	1669 [8–7279]	1482 [2–7135]	791 [2–6189]	**270 [0–5109]**	**10 [0–4156]**	312
	Citrus	1084 [15–5399]	912 [4–5339]	409 [4–4727]	**73 [0–3684]**	**0 [0–2697]**	132
	Collier	2345 [10–12,585]	1977 [4–11,765]	857 [4–10,795]	**180 [0–9139]**	**1 [0–7523]**	490
	Dixie	116 [0–536]	104 [0–527]	51 [0–476]	11 [0–420]	**0 [0–260]**	0
	Escambia	2532 [9–11,936]	2134 [4–11,449]	985 [4–9786]	**285 [0–7939]**	**7 [0–6454]**	909
	Glades	179 [35–553]	171 [23–532]	124 [7–430]	90 [1–386]	**52 [0–336]**	0
	Hendry	204 [1–1331]	174 [0–1294]	70 [0–1138]	**14 [0–890]**	**0 [0–659]**	21
	Hernando	1694 [13–7261]	1433 [4–7130]	741 [3–6422]	**203 [0–5316]**	**3 [0–3819]**	316
	Hillsborough	11,014 [60–53,152]	9456 [20–50,198]	4394 [20–39,709]	**1221 [0–31,702]**	**28 [0–25,110]**	1814
	Indian River	1458 [11–6045]	1315 [4–6008]	681 [1–5155]	250 [0–4488]	**15 [0–3647]**	207
	Leon	2348 [122–8780]	2136 [44–8379]	1333 [18–7660]	538 [0–6433]	**8 [0–4668]**	359
	Martin	1190 [7–5603]	1000 [2–5603]	534 [2–4591]	**168 [0–3756]**	**5 [0–2841]**	192
	Okeechobee	567 [27–1724]	507 [10–1635]	292 [3–1260]	194 [0–1119]	**102 [0–889]**	71
	Pasco	5933 [217–19,609]	5579 [95–19,412]	4181 [6–17,370]	2268 [0–14,532]	**265 [0–11,141]**	797
	Pinellas	7627 [43–34,894]	6051 [11–33,594]	2970 [10–28,185]	**714 [0–24,297]**	**22 [0–19,473]**	1619
	St. Johns	2415 [53–9750]	2230 [22–9414]	1428 [3–8126]	699 [0–7091]	**59 [0–6575]**	224
	St. Lucie	2578 [13–10,943]	2121 [4–10,800]	999 [4–9609]	**244 [0–7421]**	**2 [0–4878]**	354
	Sarasota	3484 [22–16,158]	2980 [7–16,019]	1699 [3–14,753]	621 [0–12,830]	**20 [0–9306]**	468
Group C	Broward	13,904 [117–70,084]	12,291 [40–67,910]	7871 [23–60,601]	**2533 [0–52,027]**	**79 [0–43,747]**	2989
	Duval	7576 [89–35,674]	6533 [27–33,558]	3209 [20–29,115]	**1038 [0–24,231]**	**71 [0–18,379]**	1758
	Lee	5133 [22–28,212]	4238 [9–28,081]	1615 [9–24,840]	**305 [0–20,604]**	**5 [0–15,306]**	873
	Manatee	3071 [9–15,744]	2533 [6–14,317]	909 [6–12,148]	**166 [0–9492]**	**4 [0–7950]**	252
	Miami-Dade	19,743 [132–87,117]	16,784 [58–83,985]	7388 [58–73,504]	**2323 [0–60,673]**	**44 [0–47,959]**	3745
	Orange	11,671 [968–41,704]	10,920 [567–40,431]	7728 [63–34,157]	4696 [0–30,214]	2796 [0–28,132]	2081
	Osceola	2714 [7–13,698]	2260 [5–12,925]	937 [5–11,248]	**255 [0–8843]**	**4 [0–6276]**	355
	Palm Beach	10,791 [218–48,492]	9534 [83–47,135]	4509 [24–40,705]	**1785 [0–34,613]**	**117 [0–29,311]**	2102
	Polk	6584 [128–27,241]	6087 [53–26,539]	4052 [13–23,713]	1559 [0–19,508]	**42 [0–13,335]**	659
	Seminole	3575 [7–17,233]	3114 [5–16,373]	1326 [5–14,693]	**356 [0–11,452]**	**14 [0–9014]**	455
	Volusia	4422 [56–19,680]	3541 [15–19,598]	1178 [10–15,709]	**244 [0–13,422]**	**7 [0–11,223]**	835
Total		168,269	146,463	78,675	29,257	4398	28,122

**Figure 5:**
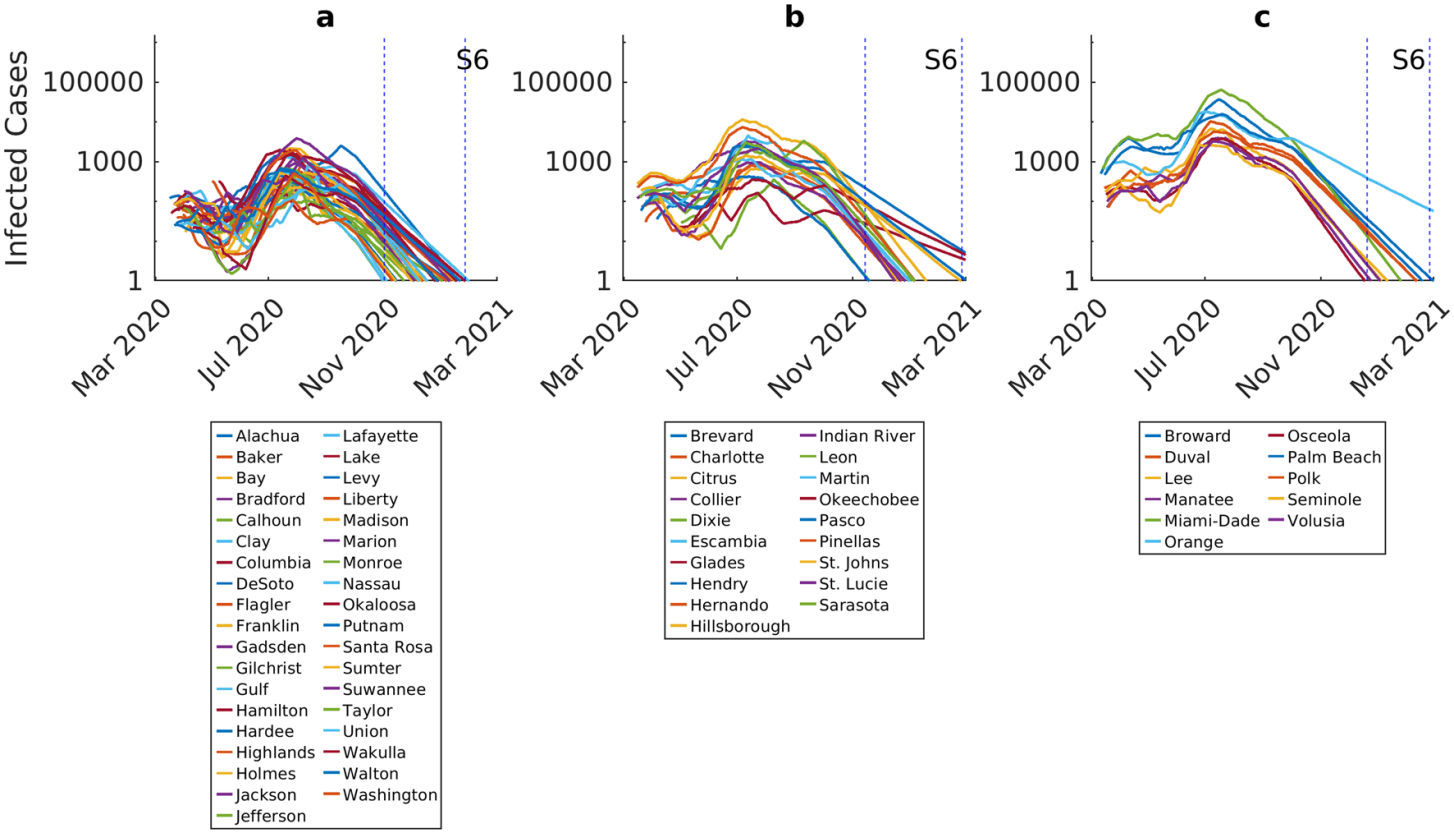
Epidemic forecasts for individual counties in the scenario where all interventions (movement restriction, social distancing, and contact tracing) are held through to March 2021 starting from October 1st 2020. Each curve represents the median prediction for a given county. The results are stratified by initial incidence growth rate (Group a < 0.05, Group b > 0.05–0.15, Group c > 0.15). The y-axis is shown in log-scale to better visualize the difference between the size of the first and second epidemic waves. Red vertical lines indicate the ranges of peak infected cases, while blue vertical lines indicate the typical ending times of the epidemic. The ending dates for the epidemic are predicted to occur between November 1st–January 26th 2021 for Group a, November 14th–February 25th 2021 for Group b, and December 20th–February 25th 2021 for Group c.

Figure 5 shows the predictions arising from the simulation of the most intensive of the social intervention scenarios (scenario 6), viz*.* maintaining social distancing, lockdown, and a 25% quarantine rate from October 1st through the end of our simulation period, March 2021. The results show that this scenario is the only one among the six scenarios investigated that would have prevented the occurrence of a 2nd wave of COVID-19 in all the modeled counties. It would also hasten the ending of the epidemic locally with all low incidence counties predicted to achieve elimination of their epidemics as early as between November 1st 2020 to January 26th 2021, whereas high incidence counties will see their corresponding epidemics ending between December 20th 2020 to February 25th 2021.

**Figure 6:**
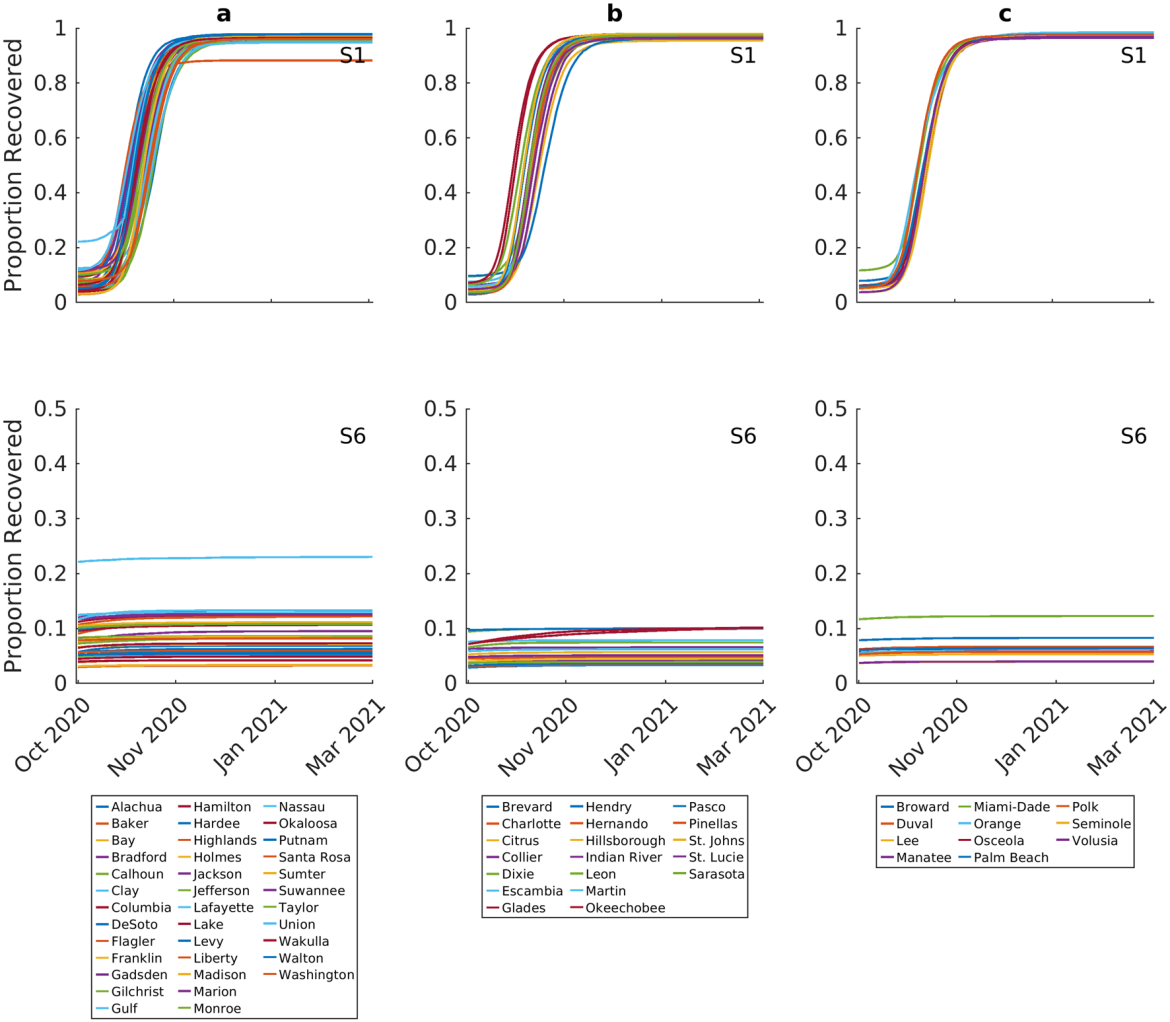
Proportion recovereds predicted over time for Scenario 1 (full release of interventions on October 1st) and Scenario 6 (lockdown, social distancing, and quarantining through to March 2021). Each curve represents the median prediction for a given county. The results are stratified by initial incidence growth rate in each county (Group a < 0.05, Group b > 0.05–0.15, Group c > 0.15).

Figure 6 depicts the proportions of the populations recovering from infection and thus developing immunity to infection through time in each county for scenarios 1 (top panel) and 6 (bottom panel). These results show that these scenarios may bring about extinction of the epidemic in each group of counties via different mechanisms. In the case of scenario 1, the epidemics are ended through the development of high levels of herd immunity (between 88 to 97%) in the community, with as expected lower population-level immunity required to bring out epidemic extinction in the lower incidence counties. By contrast, the results for scenario 6 indicate that extinction can also be brought about by instituting strong long-duration social containment measures that can reduce transmission to sufficiently low levels to bring about epidemic fade-outs. However, it is important to note that this impact comes with the cost of generating very low levels of herd immunity in each community by the end of the epidemics, raising the possibility of the inevitable resurgence of transmission should new infected individuals bring the virus back into these communities.

**Figure 7:**
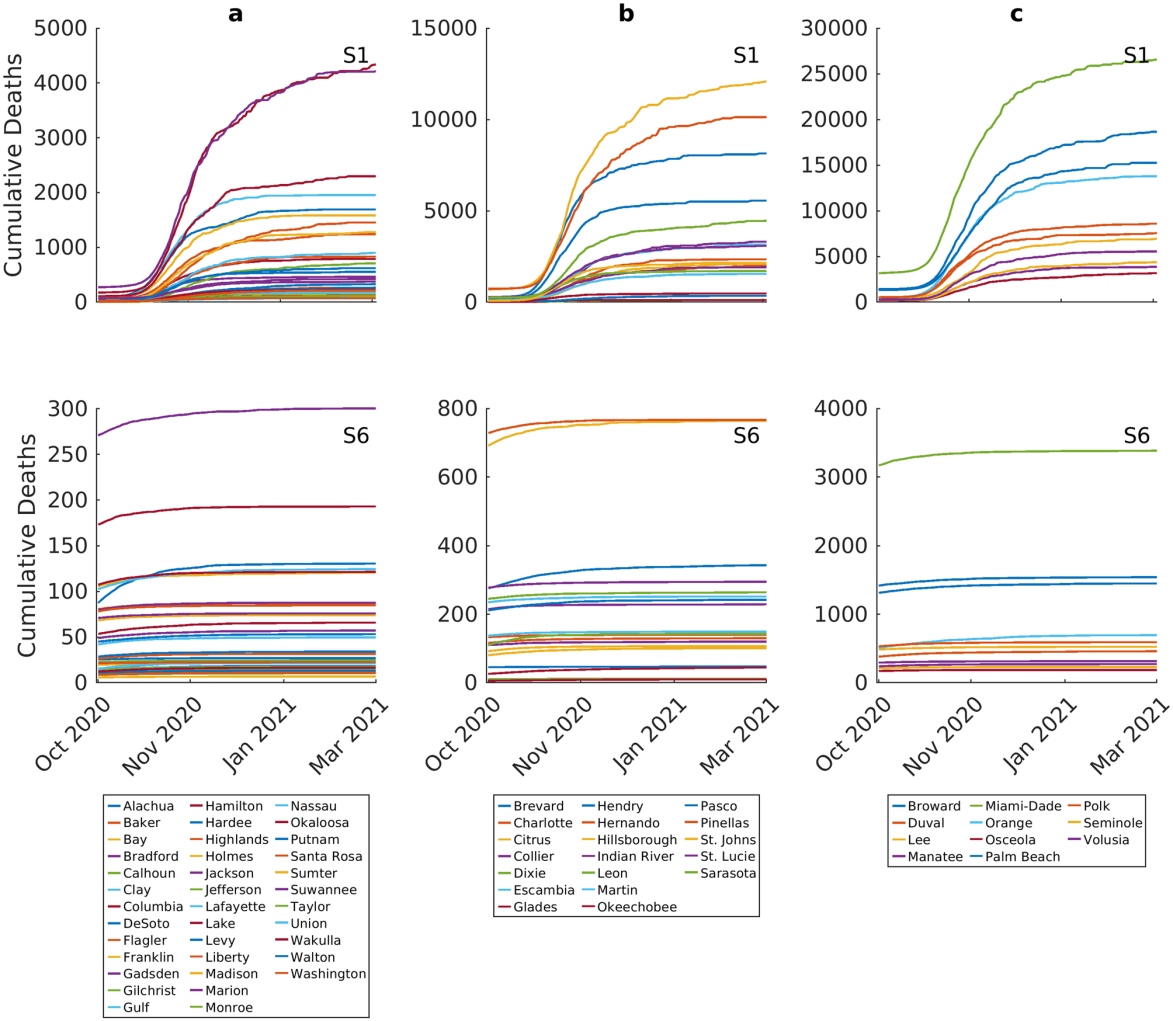
Cumulative number of deaths predicted over time for Scenario 1 (full release of interventions on October 1st) and Scenario 6 (lockdown, social distancing, and quarantining through March 2021). Each curve represents the median prediction for a given county. The results are stratified by initial incidence growth rate in each county (Group a < 0.05,  Group b > 0.05–0.15, Group c > 0.15).

Although scenario 1 can produce high levels of herd immunity (Fig. 6), it is clear, however, that this would be associated with higher death tolls than in the case of scenario 6 (Fig. 7). Cumulative predicted deaths through the entire period of these simulations (i.e. October 1st 2020 to end of March 2021) ranged from 70 to 4223 in the low incidence counties to as high as 28,130 in the high incidence counties in the case of scenario 1. These were significantly lower in the case of scenario 6 with the corresponding cumulative deaths ranging from 6 to 300 in the low incidence counties to between 185 to 3330 in the high incidence counties (Fig. 7). These findings underscore the health-economy trade-offs involved in using an approach focused on the evolution of herd immunity as opposed to one based on the use of more socially-disruptive measures for containing the present pandemic. An immediate full release of social measures is the least economically disruptive option, but results in higher cases, hospitalizations, and deaths. By contrast, implementing longer periods of social distancing measures will optimize reductions in health outcomes but will affect the working of the economy.

## Discussion

Our goals in this work were two-fold. The first was to assess if it is possible to use publicly available longitudinal infection case and human movement data to derive reasonable mathematical models of SARS-CoV-2 transmission to allow the simulation and evaluation of the course of the ongoing pandemic at the local county level in the United States. The second goal was to evaluate how the viral contagion dynamics might interact with social options for controlling COVID-19, such that the results could be used to identify those measures that will enable the safe containment of the virus in the absence of a viable vaccine. We also attempted to determine the implications of variance in virus transmission risk across smaller spatial units within a region, such as local counties, for the design of the optimal social strategies for curbing the contagion.

Developing and using reliable data-driven models for forecasting live local epidemics is challenging given the need for both the locality-specific temporal data required for updating models, and the necessity that predictions have to be made within the lead times requisite for making effective public responses^22^. Here, we have addressed this problem via the implementation of an iterative data-model assimilation-based forecasting system that acquires and processes the latest data, updates our SEIR COVID-19 model, and generates new sequential forecasts over time. Key features of the system include procedures that leverage the availability of open source API-enabled case and mortality surveillance data that are reported daily by health departments at the county level^47^, the incorporation of independently-quantified county-wide non-essential movement data to serve as an estimator of the level of population mixing through time, and the Bayesian calibration of our model on a sequential basis. An additional recent feature of the developed system is the use of a continuous analysis framework to automate the computational pipeline to handle the various stages of converting the raw data into new forecasts, including: data assembly, modelling and forecasting, and presentation of the forecasts relevant to policy makers^27,45^. This makes it possible to generate high-quality forecasts for a large number of study settings much more effectively and speedily.

We have used our modelling system to examine the effectiveness of a range of likely SARs-CoV-2 social intervention scenarios for containing the contagion at local levels, starting with investigations of the impact of the state-wide phased easing of the community lockdown that was implemented in Florida between April 3 2020 to May 4 2020 (scenario 1). Figure 2 depicts the major outcome of this policy response, viz. the inevitability of the emergence of significant 2nd waves in all counties if all other social measures, such as social distancing (mask wearing and observation of physical distancing), are also discontinued before the 1st local epidemics are fully ended. While this prediction can be considered to be as expected and so unremarkable, a striking and possibly less commented upon feature, however, is that the predicted sizes of the 2nd wave peaks or intensity of the 2nd waves will vary between counties as a positive function of variations in the size of their 1st wave peaks. This indicates that the subsequent intensity of virus transmission following the full release of all social protective measures in a community will depend fundamentally on the initial incidence at the time of epidemic establishment in a locality. It will additionally also depend on the number of infected individuals remaining in each county following the ending of the state-wide lockdown that took place before the 1st waves had been fully controlled. These findings highlight the dangers of imposing a one size fits all policy (here pertaining to the decision to ease lockdowns on a common date across Florida) for managing a spatially variable contagion.

The results pertaining to the numerical size of the 2nd peaks are shown in Table 1, and indicate that the health burden of the pandemic would also vary markedly between counties from as low as 1459 cases in Lafayette to as high as 742,898 cases for the most populous Miami-Dade county. This further underscores the fact that apart from variable resurgences in infection, spatial heterogeneity in infection burdens could also be expected if social measures are fully released in all counties. There would also be considerable variations in the course of the 2nd waves even within each incidence group (Fig. 2), although in general low incidence counties would present earlier and lower 2nd wave peaks compared to the later timings and higher peaks predicted for the corresponding medium and high incidence group of counties.

Full release of social measures would, however, as expected, result in the eventual extinction of the local epidemics in all counties, because of depletion of susceptible individuals and development of high proportions of immune individuals in each population (Fig. 6). Although this result supports the notion that permitting the development of herd immunity in populations as one way of controlling the pandemic, the predictions regarding the local and state-wide hospitalization cases (Table 2) and deaths (Fig. 7) point to the dangers of adopting such an option^8–10,12^.

Our simulations of the impacts of social control measures of varying strength and nature demonstrated overall the vital importance of continuing with these measures following phased lockdown release for containing the epidemic while waiting for more effective and less-socially disruptive pharmaceutical measures (Figs. 2, 3, 6; Table 1). According to our findings, while the two social measures investigated here, viz*.* maintaining current social distancing over a shorter (to October 14th) and longer (to November 30th) periods, and testing, contact tracing and quarantining at a moderate (25%) or higher (50%) level, would not have prevented the emergence of 2nd waves in the majority of cases, they would nonetheless delay infection peaks and reduce the numbers of patients requiring admissions to hospitals. Indeed, the inclusion of quarantine measures to March 2021, while ending social distancing by mid-October or end November 2020, for example, would have decreased the 2nd wave peak numbers of infection or hospitalizations required to over 90% of the respective 1st peak numbers in each county. Inclusion of strong contracting tracing and quarantine (scenario 5) could have led to very low levels or even interruptions of epidemic transmission and zero hospitalization cases in those counties exhibiting the lowest incidence rates among the present counties (Tables 1 and 2). This is an important outcome as it suggests that if testing and contact tracing were ramped up, then counties would have been able to lift their social distancing measures and hence reopen their economies by end of 2021 without fearing that such an option would have led to an overwhelming of their hospital capacities.

We show that the most intensive social intervention modelled in this study, viz*.* phased lockdown release, maintenance of current social distancing measures and a 25% quarantine rate all maintained from October 1st to end of March 2021, was the only social option that would have not only prevented the occurrence of a 2nd wave, but also hasten the ending of the epidemic in all counties, with extinctions predicted to be possible as early as by November 1st in some low-medium incidence counties (Fig. 5). However, like scenarios 4 and 5 but unlike the full release of social measures modelled in scenario 1, this scenario will also be marked by the low level of herd immunity that would develop in populations by the end of the local epidemics, leaving communities vulnerable to the real threat of future epidemic resurgences should the virus be re-introduced after the lifting of interventions (Fig. 6). This finding indicates that either maintaining continued vigilance and control by testing and contact tracing measures will be required to counter this prospect of epidemic resurgence in these communities over the foreseeable future, or that ultimately, control of the epidemic would only be achieved through effective vaccination of county populations. Our results show that in the latter case, vaccination rates (with a highly effective vaccine) will need to be above 85% and even above 90% in the medium–high incidence counties (Fig. 6) to accomplish the resolution of the pandemic, although note that if significant population heterogeneity underlines virus transmission within a county, much lower rates (to as low as 50%) might be sufficient to arrest the local epidemic^6,21^. This is provided that the developed immunity operates over the long-term.

Our county-level forecasts also suggest that a spatially-tailored response would be more effective at minimizing harmful effects in communities, not just in relation to health outcomes, but also in terms of minimizing the disruption to the local and global economic and other social systems. Thus, we show that while combining social distancing measures to end of December 2020 with high intensity contact tracing and quarantine to March 2021 (scenario 5) could have depressed hospitalization cases within manageable levels across virtually all county incidence groups, it would have been possible to contain the pandemic in some low and medium incidence counties with a version of this scenario (scenario 4) that implements only low intensity quarantine (Fig. 4, Tables 1 and 2). This would allow reopening of the economies of these counties earlier than for high incidence counties, lessening the economic and other social disruptions faced by the populations of these counties. Similarly, our predictions of the impact of scenario 6, in which all interventions are implemented from October 1st 2020 onwards, indicate that resolutions of the epidemic would occur significantly earlier in low incidence counties than in the case of medium and high incidence counties, suggesting that a safe reopening of the state of Florida and indeed other US states could be effectively accomplished in a geographically phased manner that takes into account county-level variations in epidemic risk explicitly. Indeed, our web-based SEIRcast COVID-19 simulation tool (https://seircast.org/) that implements our iterative data-driven continuous integration modelling framework, is designed to provide policymakers with the means to devise precisely such spatially-explicit management plans. We believe that including this spatial dimension into both models and in mitigation plans would not only provide for better predictions of the pandemic dynamics across a spatial domain, but would additionally result in significantly better overall social outcomes for state populations.

While our findings imply that social measures in general are highly effective in containing and curbing COVID-19 transmission, further work to address the rapidly changing transmission conditions affecting the pandemic and emerging interventions will undoubtedly be required to extend the applicability of the present results. Perhaps a first need is to address what impact the advent of vaccines would have on the need for continuing with the social measures investigated to allow the safe reopening of parts of the populations in Florida as early as possible. The key question here is whether NPI strategies will need to continue and indeed must remain the mainstay of our attempts to contain the contagion even with the roll out of vaccinations. Indeed, if the present vaccines can only be delivered in a phased age-targeted manner, are not perfect but instead reduces susceptibility by a fraction, and if the immunity induced is not long-term or countered by virus mutants, then there is a need to investigate how best to adapt the social measures studied here along with vaccination to bring about the containment or resolution of the pandemic effectively^54^. We are currently extending our model to include these various vaccination scenarios to address this policy question.

It is also clear going forward that we need to consider the effects that between-county movement might have on the current model predictions. While personal movement was curtailed drastically by lockdown, and the phased ending of the lockdown has led to increased movement within counties—both of which we have been able to incorporate into our model via parameterization of the within-county movement data provided by Unacast—details of inter-county movement and its reliable incorporation into our model will be required if we are to better capture the impacts of state-wide policies that are beginning to focus on lifting of all restrictions fully^55^. Recently, Unacast^52^ has begun to publish population migration data in the US using cell-phone signals, which will provide a means to address this topic.

Our current model also does not represent the age-structure and health status of the county-level populations. Partly this is an outcome of our goal to develop a modelling system that would support the generation of forecasts for the contagion in all counties of the United States based on the data presently publicly available for facilitating model configurations—and these currently lack information on these variables^47^. Extending our SEIR model to include these features, however, would allow better treatments of the exposure, risk, and transmission conditions that are likely to underlie the spatial heterogeneity in epidemic dynamics observed at the county level^18,19,31^. The addition of population structure and health composition into our current SEIR model will require deriving and adding more compartments and the applicable contact matrices^10,56,57^, but also, as noted, the configuration data for parameterizing these additions appropriately. We are currently in the process of adapting the data from the POLYMOD study^53,57^ to begin the construction of the relevant social contact matrices and parameterizations required for accomplishing these major extensions to the model. Nonetheless, it is to be noted that our data-assimilation approach to estimating the transmission rate in each country (both the median and range of values from the ensemble of best-fit models) implicitly does allow capture of the contributions of age-structural and other differences in transmission between counties, suggesting we have been able to approximate the impacts of this factor to a reasonable degree on the results presented here.

Our sequential data-assimilation framework, while allowing the incorporation of longitudinal changes in transmission conditions into the model, has the outcome, as for all dynamical models, that prediction error will increase the further out of sample forecasts are made^22–25,27,29^. While we have attempted to reduce forecast errors by model fitting to two sets of variables (infection cases and deaths), obtaining new data on other currently latent states (e.g. the fraction of asymptomatic infected cases) would offer better constraining of parameters and hence forecast variance. However, this must be balanced by appropriately addressing the effects of parameter degeneracy and sample impoverishment, which would impact the ability of the model to fit novel data as transmission conditions change drastically over the near future^37,58–60^. We have used a resampling approach whereby at each sequential updating point, we have blended in 25% random samples from initial priors to the posteriors obtained during the uptake made a time step (every 2-weeks) previously to keep forecast error below 20% to address this problem in the simulations reported here. However, future work might need to consider the development of appropriate adaptive approaches developed in the field of particle filtering^61^ to resolve this problem more effectively. Regardless, we note that while our forecasts beyond 2 weeks ahead could attain variances as high as 40%, and so can affect the peak sizes and extinction dates reported here, this will have lesser impacts on the conclusions reached regarding the comparative outcomes of the interventions investigated in this study.

## Methods

### Epidemic model

**Figure 8:**
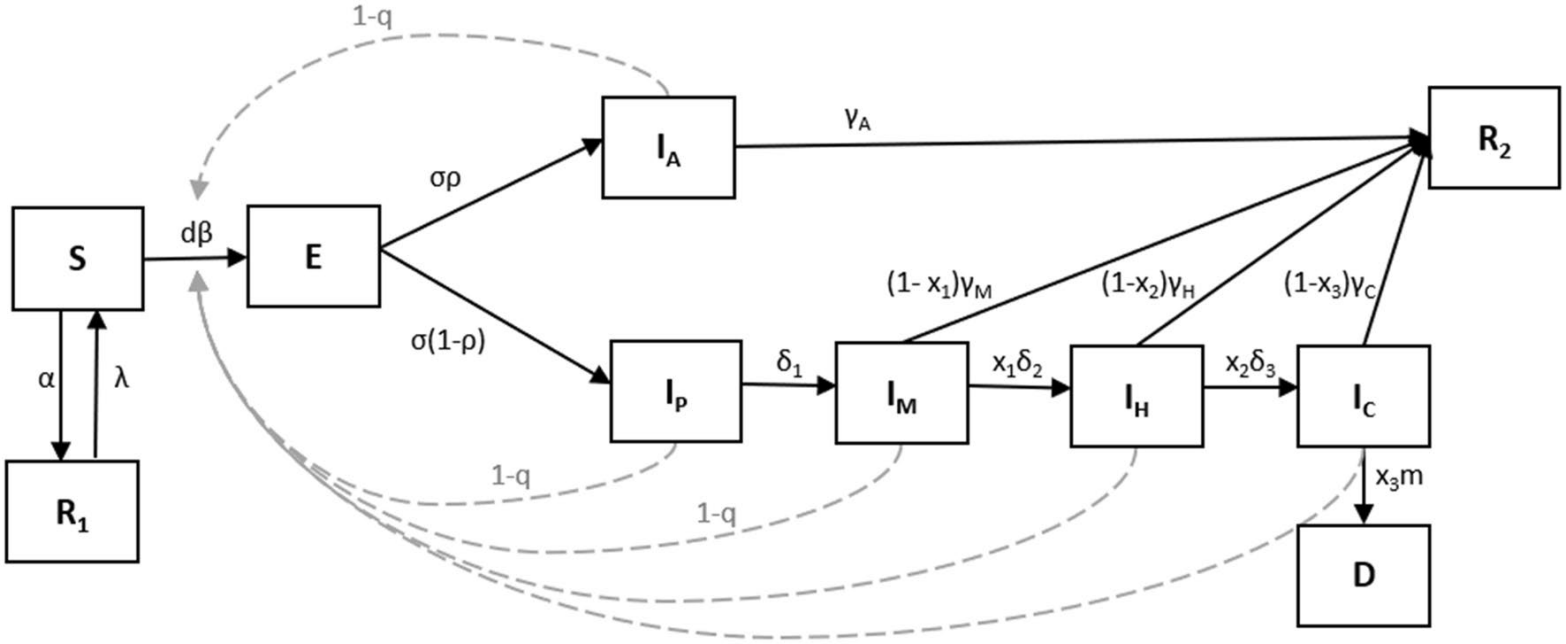
Compartmental model structure. Boxes or compartments represent host categories related to infection status, solid lines represent movement between compartments, and dashed arrows depict the states contributing to transmission. Full details of model structure and the full set of differential equations and parameters driving the model are given in Supplementary Table S3.

We simulated the ongoing SARS-CoV-2 outbreaks at the county level using a variation of the SEIR model. The model compartments and transitions are shown in Fig. 8. Full equations are also given in Supplementary Material. We assume each county is a closed population and ignore demographic changes such that the total population size remains constant. The population is divided into compartments representing various infection stages: susceptible (S), susceptible but removed from the transmission process via lockdown policies (R_1_), exposed (E), infectious asymptomatic (I_A_), infectious pre-symptomatic (I_P_), infectious with mild symptoms (I_M_), infectious with severe symptoms requiring hospitalization (I_H_), infectious with severe symptoms requiring intensive care including ventilation (I_C_), recovered and immune (R_2_), and deceased (D). The model equations describing the transitions in and out of each class are given in the Supplementary Material along with a description of all the model parameters and their prior values (Supplementary Table S3). Note the model considers the fraction of the population classified in each compartment (all compartments sum to 1), which is then scaled to the appropriate county population size to get counts for each compartment.

### Data

To calibrate the model to the local county setting, we fitted the SEIR model sequentially (see below) to cumulative confirmed case and deaths data assembled from the start of the epidemic at the county level and published for public access by the Johns Hopkins University Coronavirus Resource Center^47^. The county population sizes are also made available via this database, which we use to scale the model predictions (see above). Hospital bed capacity in each county are provided by the Agency for Health Care Administration for the State of Florida accessed on April 5th^62^. A 7-day moving average is applied to the case and death data to smooth out testing irregularities.

### Estimation of initial epidemic growth rate

The initial incidence growth rate, *τ*, was estimated by fitting a log-linear model to the daily new cases reported during the early exponential phase (the first 4 weeks generally) of the epidemic curve observed in each county^63^. The values estimated for *τ* in each county were used to stratify the counties in Florida into each of initially low (< 0.05), medium (> 0.05 to < 0.15) and high (> 0.15) incidence or epidemic groups.

### Bayesian melding data assimilation

We used a Monte-Carlo-based Bayesian melding framework to undertake the sequential updating of the model to the cumulative case and death data^40,64,65^. We began by first defining uniform prior distributions for each of the model parameters based on current understanding of SARS-CoV-2 transmission and disease characteristics. These initial parameter priors and relevant references are given in Supplementary Table S3. Note that the number of initial infected cases at the start of the simulation period is sampled as the parameter E0 (Supplementary Fig. S4), the number of exposed cases when the first cases began to be confirmed. We consider the start of the epidemic in each county to be when there are at least 10 cases reported. At this point, we sampled N = 50,000 parameter vectors from the initial priors and simulate the outbreak for 14 days forward. The resulting 50,000 model predictions of the epidemic are then compared to the confirmed case and death data observed during the 14-day forecast period using a modified root-mean-square error distance metric that normalizes a traditional RMSE by the standard deviations of these data. This facilitated the combination of prediction errors with respect to case and death data together despite their different orders of magnitude:

$$MRMSE=\sqrt{\frac{1}{2n}{\sum }_{i=1}^{n}\frac{{\left({\widehat{y}}_{i}-{y}_{i}\right)}^{2}}{std(\widehat{y})}+\frac{{\left({\widehat{x}}_{i}-{x}_{i}\right)}^{2}}{std(\widehat{x})}},$$

where *n* is the number of time points over which to compare the model predictions to data, *ŷ*_*i*_ is the model-predicted confirmed case data on a given date *i*, and *y*_*i*_ is the observed confirmed case count for the same date, *x̂*_*i*_ is the model-predicted death data on a given date *i*, and *x*_*i*_ is the observed death count for the same date. Based on this performance metric, the best-fitting 500 parameter vectors are retained as the most likely parameter sets to describe the local outbreak during the chosen 14-day window. For simulating the epidemic for the next 14 day period, another 50,000 parameters sets are sampled of which 75% are randomly sampled from the posterior distribution of the most recent 14 day window, while another 25% are sampled from the initial parameter priors to avoid sample depletion^59,60^. These set of blended parameter vectors are used to sequentially select the best-fitting models over time, and are used to forecast the impacts of the interventions described above. Different fitting windows were tried, and a 14-day window was found to be long enough to be computationally feasible for the entire dataset for all counties, while being short enough to capture the changing epidemic behavior and keep forecast error consistently low (below 20%).

The best-fitting parameter vectors are used to simulate future scenarios. The forecasts allow for the prediction of future waves of infection, which are defined as a sustained positive growth rate of cases, leading to a maximum. The subtleties of identifying the exact timing of waves due to trivial oscillations in data has been explored in other works^66^.

### Simulating interventions

We used the latest sequentially fitted model in each county to simulate the impacts of different social intervention scenarios on the course of the outbreak in the future (beyond October 1st 2020). We simulated six different scenarios, which are outlined graphically in Supplementary Fig. S3. Scenario 1 represents the least aggressive option where lockdown and social distancing measures (like modified behavior, physical distancing, mask wearing, and increased sanitization) are fully lifted after September 30th. Scenario 2 maintains lockdown in addition to keeping social distancing measures in place for 2 weeks from October 1st to October 14th. We consider scenarios 1 and 2 to mimic the State of Florida’s state reopening plan (https://floridahealthcovid19.gov/plan-for-floridas-recovery/). Scenario 3 extends the social interventions (lockdown plus social distancing measures) by maintaining it over a longer 8 week period to November 30th. Scenarios 4 and 5 represent maintaining current social distancing and movement restrictions through the end of the year (December 2020) in addition to implementing contact tracing and quarantine efforts at either low (*q* = 0.25) or high (*q* = 0.50) intensity, respectively, from October 1st to end of March 2021. Finally, Scenario 6 represents the most intense intervention scenario, viz. maintaining social distancing, lockdown, and low quarantine starting from October 1st through to the end of March 2021. We considered this to be the most intense intervention because we maintain all three social interventions for the longest period of time. We implemented a low quarantine effort in this scenario to represent an incremental increase in the intensity of interventions relative to Scenario 5. This allowed us to also investigate if this was sufficient given the longer period of interventions to have the biggest impact on the pandemic among all the scenarios investigated in this paper. Outbreaks were simulated under these conditions and the predicted number of cases are compared between each scenario and county group. The numbers of hospitalized individuals (I_H_ + I_C_) are also forecasted to evaluate the potential resource needs under each scenario.

The effect of statewide lockdown measures in these simulations is implemented by adding a distinct susceptible class which is assumed to not contribute to disease transmission (R_1_). The proportion of this class that complies with strict stay-at-home orders is controlled through the ratio of parameters *α* and *λ*. This ratio is informed by the fraction of non-essential trips made by the population in each county as estimated by Unacast based on analyses of GPS mobility data^52^. Mobility data has been used as a predictive tool across many domains—it has been used to build mobility networks^67^, study the relationship between mobility and financial market performance^68^, and understand which mobility patterns lead to an increased risk of death due to COVID-19 infection^69^. We interpret the reduction in such non-essential trips from prior to the lockdown as a proxy for the proportion of the susceptible population remaining unexposed in a county during any time of a simulation—both during the lockdown and after lockdown measures were lifted. Note that, additionally, incorporating this fraction of protected susceptibles into the model by this means also allows us to address the question of self-isolation by individuals indirectly. Social distancing measures are modeled as a reduction in transmissibility of the pathogen through the parameter *d* and is primarily based on the effectiveness of masks against transmission of similar diseases^70^. Although the parameter *d* could capture the effect of lockdown in addition to social distancing measures, we decided to model the population under lockdown separately via the use of the independent Unacast data to retain our ability to undertake investigations, if required, of the relative impacts of these measures on the course of the pandemic in future simulations. Quarantine of infectious cases through contact tracing and/or testing is modeled simply as a proportion, *q*, of I_A_, I_P_, and I_M_ as not contributing to transmission as a result of being detected and made to isolate themselves at home.

## Supplementary Information

Supplementary Information.

## Data Availability

All data analyzed during this study are included in this published article and its supplementary information files. Code for sequentially calibrating and running the SEIR model is available at https://github.com/EdwinMichaelLab/COVID-SEIR-Paper.
