# Supplementary material for: Iterative data-driven forecasting of the transmission and management of SARS-CoV-2/COVID-19 using social interventions at the county-level
Source: Sci Rep. 2022 Jan 18;12:890. doi: 10.1038/s41598-022-04899-4 (PMC8766467; doi:10.1038/s41598-022-04899-4)
Supplement: Supplementary file 1 — Supplementary Information. [file 41598_2022_4899_MOESM1_ESM.docx]

Supplementary Information for

# Iterative near-term forecasting of the transmission and management of SARS-CoV-2/COVID-19 using social interventions at the county-level

Ken Newcomb^1^, Morgan E. Smith^2^, Rose E. Donohue^2^, Sebastian Wyngaard^3^, Caleb Reinking^3^, Christopher R. Sweet^3^, Marissa J. Levine^4^, Thomas R. Unnasch^1^, Edwin Michael^1*^

^1^ Center for Global Health Infectious Disease Research, University of South Florida, Tampa, FL, USA

^2^ Department of Biological Sciences, University of Notre Dame, Notre Dame, IN, USA

^3^ Center for Research Computing, University of Notre Dame, Notre Dame, IN, USA

^4^ Center for Leadership in Public Health Practice, University of South Florida, Tampa, FL

* Edwin Michael

emichael@nd.edu

**This PDF file includes:**

Supplementary text

Figures S1 to S4

Tables S1 to S3

SI References

# Supplementary text

The ordinary differential equations of our compartmental SARS-CoV-2 transmission model are:

$$\begin{matrix} \frac{dS(t)}{dt}=-d\beta S(t)\left[\left(1-q\right)\left(I_{A}(t)+I_{P}(t)+I_{M}(t)\right)+I_{H}(t)+I_{C}(t)\right]-\alpha S(t)+\lambda R_{1} \\ \frac{dE(t)}{dt}=d\beta S(t)\left[\left(1-q\right)\left(I_{A}(t)+I_{P}(t)+I_{M}(t)\right)+I_{H}(t)+I_{C}(t)\right]-\sigma\rho E(t)-\sigma(1-\rho)E(t) \\ \frac{dI_{A}(t)}{dt}=\sigma\rho E(t)-\gamma_{A}I_{A}(t) \\ \frac{dI_{P}(t)}{dt}=\sigma(1-\rho)E(t)-\delta_{1}I_{P}(t) \\ \frac{dI_{M}(t)}{dt}=\delta_{1}I_{P}(t)-x_{1}\delta_{2}I_{M}(t)-(1-x_{1})\gamma_{M}I_{M}(t) \\ \frac{dI_{H}(t)}{dt}=x_{1}\delta_{2}I_{M}(t)-x_{2}\delta_{3}I_{H}(t)-(1-x_{2})\gamma_{H}I_{H}(t) \\ \frac{dI_{C}(t)}{dt}=x_{2}\delta_{3}I_{H}(t)-(1-x_{3})\gamma_{C}I_{C}(t)-x_{3}mI_{C}(t) \\ \frac{dD(t)}{dt}=x_{3}mI_{C}(t) \\ \frac{dR_{1}(t)}{dt}=\alpha S(t)-\lambda R_{1} \\ \frac{dR_{2}(t)}{dt}=\gamma_{A}I_{A}(t)+(1-x_{1})\gamma_{M}I_{M}(t)+(1-x_{2})\gamma_{H}I_{H}(t)+(1-x_{3})\gamma_{C}I_{C}(t) \\ Q(t)=q(I_{A}(t)+I_{P}(t)+I_{M}(t)) \end{matrix}$$

The population is divided into compartments representing infection status: susceptible (S), susceptible but removed from the transmission process via lockdown policies (R_1_), exposed (E), infectious asymptomatic (I_A_), infectious pre-symptomatic (I_P_), infectious with mild symptoms (I_M_), infectious with severe symptoms requiring hospitalization (I_H_), infectious with severe symptoms requiring intensive care including ventilation (I_C_), recovered and immune (R_2_), and deceased (D). We also model a population which is quarantined (Q) after testing and/or contact tracing as a fraction of the relatively mild infectious classes I_A_, I_P_, and I_M_. All model parameters are described in Table S3.


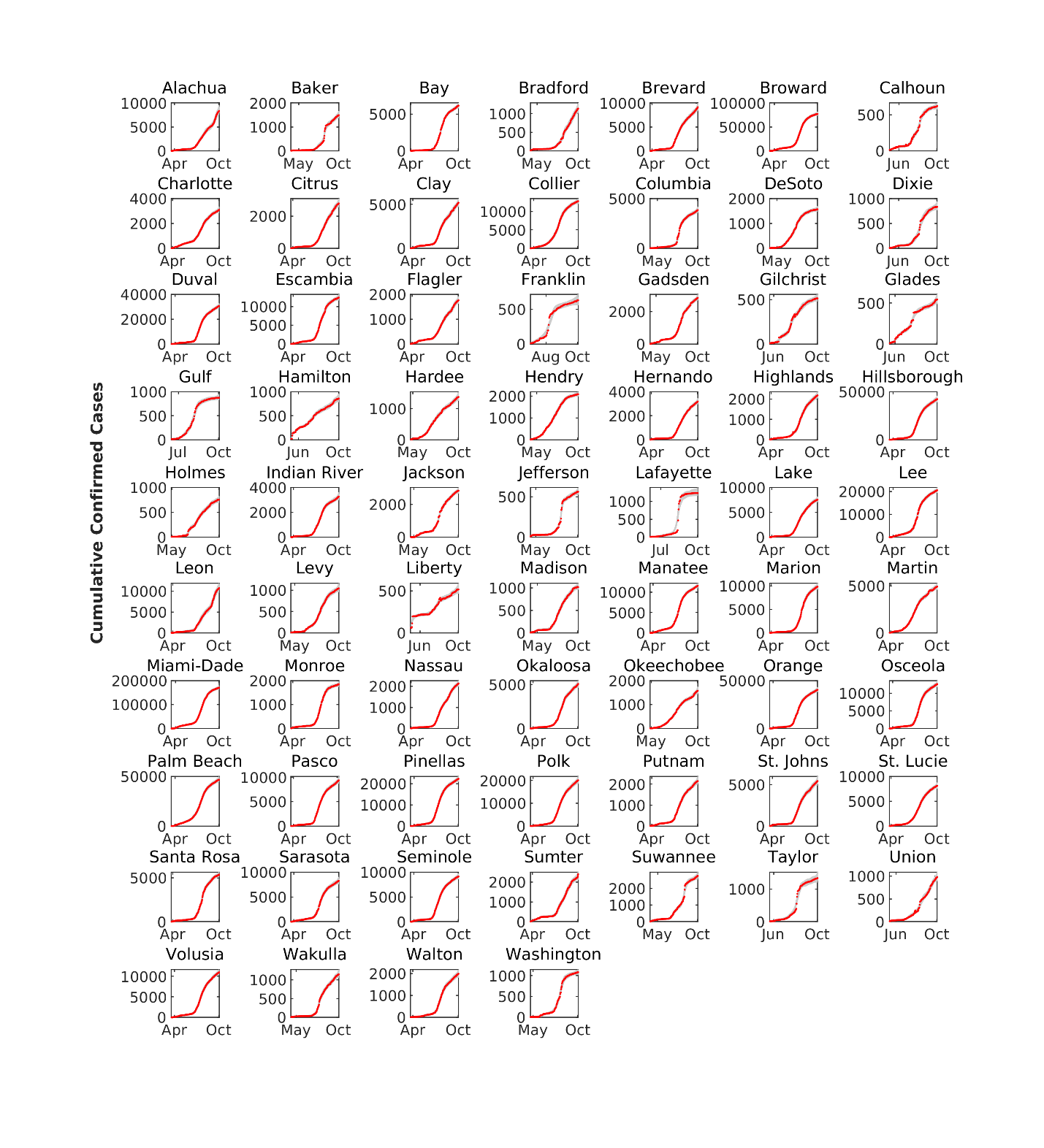


Figure S1. Model fits compared to confirmed cumulative case data in all 67 Florida counties**.** Gray curves represent county-specific model predictions, and red points represent confirmed case data obtained from Johns Hopkins University ^38^. Fitting was started after at least 10 confirmed cases were reported.


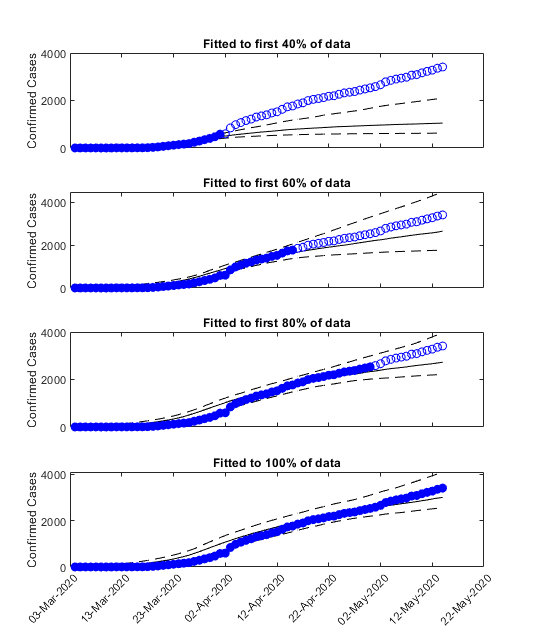


Figure S2. Iterative fits to segments of data. Solid points show which data points were used to calibrate the model and open circles show which data points were left out for validation purposes.


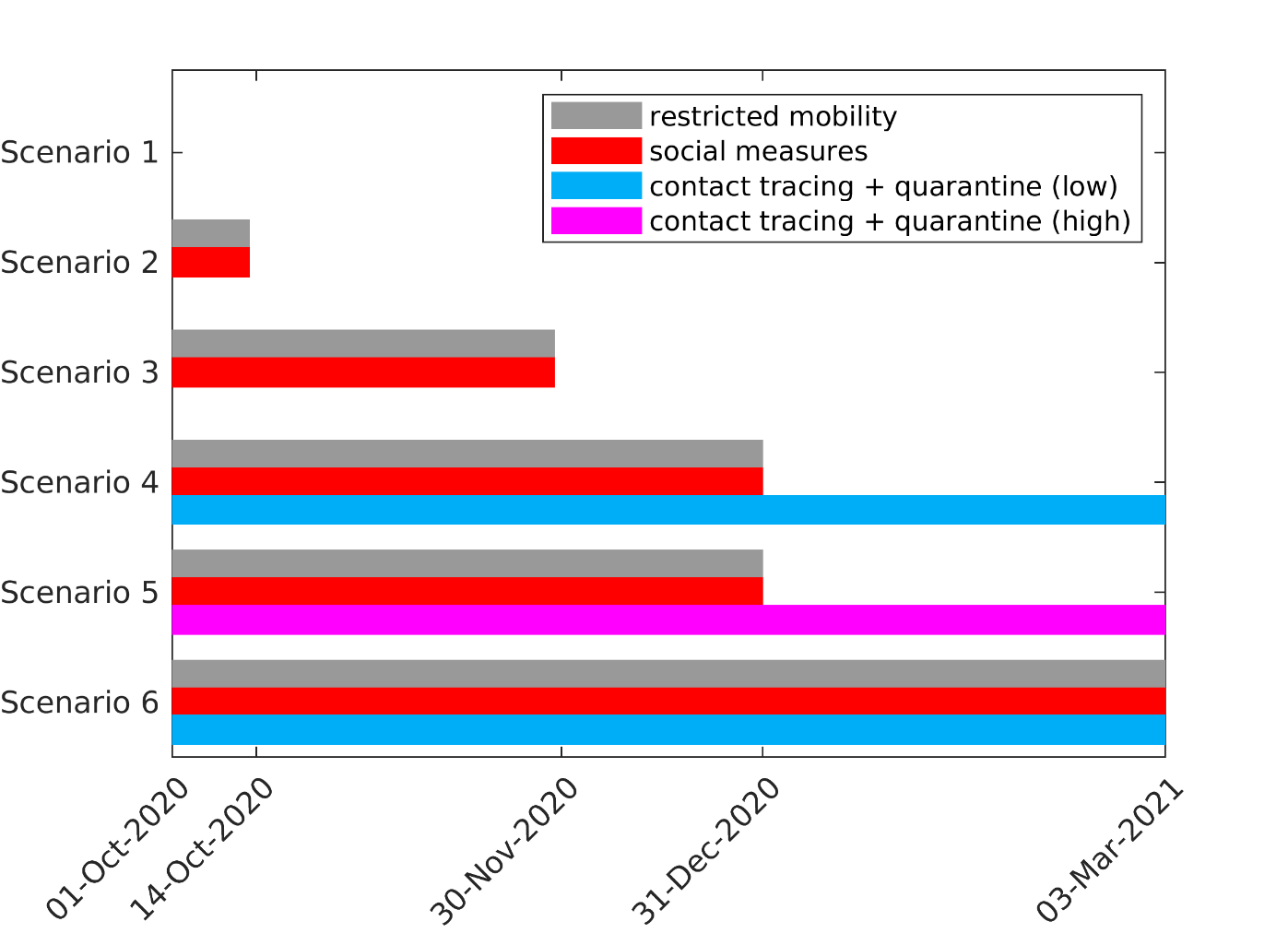


Figure S3. Description of modeled intervention scenarios. The colored bars indicate the duration of lockdown, social distancing measures, and contact tracing and quarantine efforts.

Scenario 1 represents the least aggressive option where lockdown and social distancing measures (which includes everything from modified behavior, physical distancing, mask wearing, and increased sanitization) are fully lifted after September 30^th^. Scenario 2 maintains lockdown in addition to keeping social distancing measures in place for 2 weeks from October 1^st^ to October 14^th^. We consider scenarios 1 and 2 to mimic the State of Florida’s state reopening plan (<https://floridahealthcovid19.gov/plan-for-floridas-recovery/>). Scenario 3 extends the social distancing interventions (lockdown plus social distancing measures) by maintaining it over a longer 8 week period to November 30th. Scenarios 4 and 5 represent maintaining current social distancing and movement restrictions through the end of the year (December 2020) in addition to implementing contact tracing and quarantine efforts at either low (*q* = 0.25) or high (*q* = 0.50) intensity, respectively, from October 1^st^ to end of March 2021. Finally, Scenario 6 represents the most socially intense intervention scenario, *viz*. maintaining social distancing, lockdown, and low quarantine starting from October 1^st^ through to the end of March 2021.


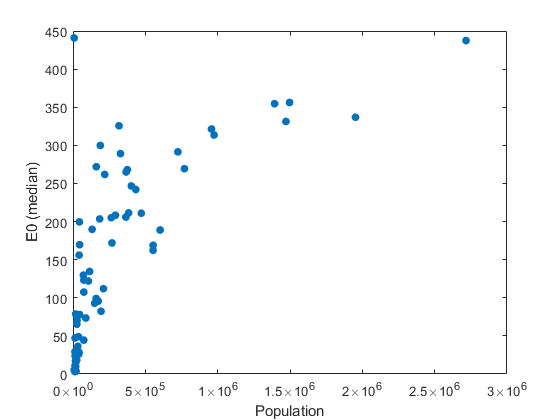


Figure S4. Relationship between the fitted value of E0 (initial exposed cases at start of simulation) and county population size.

Table S1. List of Florida counties and their population sizes.

| County | Population | County | Population |
| --- | --- | --- | --- |
| Alachua | 269043 | Lee | 770577 |
| Baker | 29210 | Leon | 293582 |
| Bay | 174705 | Levy | 41503 |
| Bradford | 28201 | Liberty | 8354 |
| Brevard | 601942 | Madison | 18493 |
| Broward | 1952778 | Manatee | 403253 |
| Calhoun | 14105 | Marion | 365579 |
| Charlotte | 188910 | Martin | 161000 |
| Citrus | 149657 | Miami-Dade | 2716940 |
| Clay | 219252 | Monroe | 74228 |
| Collier | 384902 | Nassau | 88625 |
| Columbia | 71686 | Okaloosa | 210738 |
| DeSoto | 38001 | Okeechobee | 42168 |
| Dixie | 16826 | Orange | 1393452 |
| Duval | 957755 | Osceola | 375751 |
| Escambia | 318316 | Palm Beach | 1496770 |
| Flagler | 115081 | Pasco | 553947 |
| Franklin | 12125 | Pinellas | 974996 |
| Gadsden | 45660 | Polk | 724777 |
| Gilchrist | 18582 | Putnam | 74521 |
| Glades | 13811 | Santa Rosa | 184313 |
| Gulf | 13639 | Sarasota | 433742 |
| Hamilton | 14428 | Seminole | 471826 |
| Hardee | 26937 | St. Johns | 264672 |
| Hendry | 42022 | St. Lucie | 328297 |
| Hernando | 193920 | Sumter | 132420 |
| Highlands | 106221 | Suwannee | 44417 |
| Hillsborough | 1471968 | Taylor | 21569 |
| Holmes | 19617 | Union | 15237 |
| Indian River | 159923 | Volusia | 553284 |
| Jackson | 46414 | Wakulla | 33739 |
| Jefferson | 14246 | Walton | 74071 |
| Lafayette | 8422 | Washington | 25473 |
| Lake | 367118 |  |  |

Table S2. Median and range of social distancing parameter (d) and lockdown fraction over the last 14 days of data, using the 500 best-fitting models. Shaded cells denote the counties for which state-level mobility data was used to estimate population movement restrictions due to lack of county-level data.

| Class | County | Median, d | Range, d | Median, lockdown fraction | Range, lockdown fraction |
| --- | --- | --- | --- | --- | --- |
| Group A | Alachua | 0.233 | 0.104-0.396 | 0.299 | 0.076-0.35 |
|  | Baker | 0.228 | 0.102-0.396 | 0.010 | 0.01-0.105 |
|  | Bay | 0.267 | 0.103-0.398 | 0.334 | 0.219-0.389 |
|  | Bradford | 0.231 | 0.101-0.394 | 0.032 | 0.002-0.146 |
|  | Calhoun | 0.236 | 0.1-0.399 | 0.334 | 0.219-0.389 |
|  | Clay | 0.251 | 0.103-0.397 | 0.274 | 0.192-0.347 |
|  | Columbia | 0.233 | 0.101-0.397 | 0.010 | 0.01-0.166 |
|  | DeSoto | 0.230 | 0.1-0.398 | 0.220 | 0.057-0.439 |
|  | Flagler | 0.214 | 0.101-0.398 | 0.220 | 0.104-0.256 |
|  | Franklin | 0.259 | 0.102-0.399 | 0.334 | 0.219-0.389 |
|  | Gadsden | 0.224 | 0.103-0.398 | 0.189 | 0.035-0.416 |
|  | Gilchrist | 0.221 | 0.103-0.399 | 0.012 | 0.01-0.365 |
|  | Gulf | 0.240 | 0.103-0.397 | 0.334 | 0.219-0.389 |
|  | Hamilton | 0.259 | 0.103-0.398 | 0.334 | 0.219-0.389 |
|  | Hardee | 0.264 | 0.101-0.398 | 0.217 | 0.121-0.316 |
|  | Highlands | 0.260 | 0.101-0.399 | 0.402 | 0.301-0.498 |
|  | Holmes | 0.229 | 0.102-0.399 | 0.089 | 0.01-0.476 |
|  | Jackson | 0.261 | 0.101-0.398 | 0.334 | 0.219-0.389 |
|  | Jefferson | 0.237 | 0.101-0.398 | 0.023 | 0.01-0.314 |
|  | Lafayette | 0.243 | 0.104-0.4 | 0.334 | 0.219-0.389 |
|  | Lake | 0.220 | 0.101-0.399 | 0.268 | 0.169-0.36 |
|  | Levy | 0.210 | 0.102-0.397 | 0.042 | 0.01-0.25 |
|  | Liberty | 0.305 | 0.108-0.398 | 0.334 | 0.219-0.389 |
|  | Madison | 0.245 | 0.1-0.398 | 0.353 | 0.293-0.481 |
|  | Marion | 0.254 | 0.102-0.399 | 0.222 | 0.105-0.3 |
|  | Monroe | 0.245 | 0.101-0.4 | 0.334 | 0.219-0.389 |
|  | Nassau | 0.232 | 0.1-0.397 | 0.267 | 0.12-0.324 |
|  | Okaloosa | 0.247 | 0.103-0.399 | 0.334 | 0.219-0.389 |
|  | Putnam | 0.244 | 0.1-0.398 | 0.124 | 0.01-0.238 |
|  | Santa Rosa | 0.227 | 0.101-0.398 | 0.334 | 0.219-0.389 |
|  | Sumter | 0.248 | 0.101-0.398 | 0.406 | 0.285-0.461 |
|  | Suwannee | 0.259 | 0.101-0.399 | 0.334 | 0.219-0.389 |
|  | Taylor | 0.240 | 0.102-0.398 | 0.010 | 0.01-0.176 |
|  | Union | 0.244 | 0.103-0.399 | 0.334 | 0.219-0.389 |
|  | Wakulla | 0.219 | 0.101-0.4 | 0.107 | 0.01-0.219 |
|  | Walton | 0.252 | 0.1-0.4 | 0.334 | 0.219-0.389 |
|  | Washington | 0.247 | 0.101-0.4 | 0.334 | 0.219-0.389 |
| Group B | Brevard | 0.262 | 0.102-0.396 | 0.232 | 0.049-0.317 |
|  | Charlotte | 0.256 | 0.103-0.395 | 0.413 | 0.318-0.476 |
|  | Citrus | 0.218 | 0.101-0.398 | 0.239 | 0.049-0.349 |
|  | Collier | 0.242 | 0.103-0.4 | 0.389 | 0.235-0.473 |
|  | Dixie | 0.232 | 0.102-0.398 | 0.334 | 0.219-0.389 |
|  | Escambia | 0.241 | 0.103-0.398 | 0.359 | 0.112-0.873 |
|  | Glades | 0.314 | 0.104-0.399 | 0.334 | 0.219-0.389 |
|  | Hendry | 0.234 | 0.103-0.399 | 0.241 | 0.075-0.322 |
|  | Hernando | 0.245 | 0.102-0.399 | 0.299 | 0.19-0.398 |
|  | Hillsborough | 0.248 | 0.101-0.398 | 0.330 | 0.249-0.418 |
|  | Indian River | 0.258 | 0.101-0.396 | 0.341 | 0.202-0.412 |
|  | Leon | 0.240 | 0.105-0.4 | 0.390 | 0.261-0.444 |
|  | Martin | 0.257 | 0.1-0.399 | 0.303 | 0.158-0.374 |
|  | Okeechobee | 0.283 | 0.1-0.397 | 0.168 | 0.01-0.357 |
|  | Pasco | 0.254 | 0.102-0.395 | 0.247 | 0.142-0.337 |
|  | Pinellas | 0.258 | 0.101-0.399 | 0.369 | 0.251-0.434 |
|  | St. Johns | 0.250 | 0.1-0.398 | 0.295 | 0.166-0.403 |
|  | St. Lucie | 0.243 | 0.1-0.397 | 0.290 | 0.158-0.392 |
|  | Sarasota | 0.260 | 0.104-0.4 | 0.383 | 0.239-0.464 |
| Group C | Broward | 0.244 | 0.102-0.391 | 0.395 | 0.312-0.465 |
|  | Duval | 0.254 | 0.101-0.393 | 0.282 | 0.16-0.334 |
|  | Lee | 0.253 | 0.102-0.399 | 0.367 | 0.21-0.413 |
|  | Manatee | 0.255 | 0.1-0.397 | 0.332 | 0.167-0.409 |
|  | Miami-Dade | 0.257 | 0.101-0.396 | 0.397 | 0.348-0.472 |
|  | Orange | 0.250 | 0.101-0.396 | 0.449 | 0.365-0.507 |
|  | Osceola | 0.263 | 0.107-0.398 | 0.424 | 0.337-0.482 |
|  | Palm Beach | 0.240 | 0.102-0.397 | 0.383 | 0.301-0.45 |
|  | Polk | 0.245 | 0.1-0.396 | 0.265 | 0.173-0.354 |
|  | Seminole | 0.249 | 0.102-0.398 | 0.287 | 0.194-0.34 |
|  | Volusia | 0.246 | 0.101-0.397 | 0.356 | 0.204-0.453 |
| State of Florida | | 0.245 | 0.210-0.314 | 0.334 | 0.01-0.449 |
| Kruskal – Wallis test | | p < 0.001 | | p < 0.001 | |

Table S3. Model parameters definitions and prior distributions (reflects best understanding as of April 2020).

| Parameter | Definition | Prior range | Units/notes | Published values | References |
| --- | --- | --- | --- | --- | --- |
| β | Infection transmission rate | 0.1428 – 1.5 | Estimated as R0*gamma in SIR model | R0 = 2-6  β = 0.6 – 1.7 | (1-4) |
| L | Lockdown ratio | 3-5 | Ratio of population under lockdown to susceptible population | - | - |
| α | Rate of entering lockdown | 2.0 (fixed) | Controls how quickly lockdown is enforced, linked to lockdown ratio and lambda | Model structure proposed by Peng | (2) |
| λ | Rate of leaving lockdown | = alpha/L |  | Model structure proposed by Jiwei | (5) |
| σ | Rate of moving from exposed class to infectious class | 0.16 – 0.5 | 1/σ is the latent period; assumed 2-6 days | 2.2-6 days latent period | (4) |
| p | Proportion of exposed who become asymptomatic | 0.1 – 0.3 |  |  | (6-8) |
| γ_A_ | Recovery rate of asymptomatic cases | 0.125 – 0.33 | 1/γ_A_ is the infectious period; assumed 3-8 days | 3-14 days infectious period, most <= 8 days | (3, 4, 9-11) |
| γ_M_ | Recovery rate of cases with mild symptoms | 0.125 – 0.33 | 1/γ_M_ is the infectious period; assumed 3-8 days | 3-14 days infectious period, most <= 8 days | (3, 4, 9-11) |
| γ_H_ | Recovery rate of cases with severe symptoms requiring hospitalization | 0.125 – 0.33 | 1/γ_H_ is the infectious period of severe cases; assumed 3-8 days | 3-14 days infectious period, most <= 8 days | (3, 4, 9-11) |
| γ_C_ | Recovery rate of cases with severe symptoms requiring intensive care | 0.125 – 0.33 | 1/γ_C_ is the infectious period; assumed 3-8 days | 3-14 days infectious period, most <= 8 days | (3, 4, 9-11) |
| δ_1_ | Rate of moving from presymptomatic class to mild symptomatic | 0.05 – 1 | 1/time from start of infectious period to illness onset; assume 1-20 days | Latent period: 2-6 days  Incubation period: 2-12 days | (4, 12-14) |
| δ_2_ | Rate of moving from mild case to hospitalized class | 0.06 – 0.25 | 1/time from illness onset to  hospitalization; assume 4-15 days | 4-15 days | (14-16) |
| δ_3_ | Rate of moving from hospitalized class to ICU | 0.09 – 1 | 1/time from hospitalization to ICU; assume 1-11 days | Illness onset to ICU: 6-15 days  Illness onset to hospital: 4-15 days | (14-16) |
| m | Mortality rate of ICU class | 0.08 – 0.25 | 1/time from ICU to death | 4-12 days | (16) |
| ε | Proportion of symptomatic cases that are not reported | 0.1 – 0.3 | Assume 10-30% of symptomatic cases are not getting tested | Diagnostic rate of symptomatic 1/9 -1/3; Diagnosis rate overall 25% | (5, 17) |
| x_1_ | Proportion of mild cases that progress to hospital | 0.05 – 0.3 | 5-30% of mild cases are hospitalized | 5%, 20.7-31.4% | (18, 19) |
| x_2_ | Proportion of hospital cases that progress to ICU | 0.2 – 0.3 | 20-30% of hospitalized cases require an ICU | 26-30% | (15, 16, 19, 20) |
| x_3_ | Proportion of ICU cases that die | 0.2 – 0.8 | Proportion of ICU cases that die |  | (16, 20, 21) |
| d | Reduction in transmission due to social distancing, face masks, etc. | 0.1 – 0.4 |  | Masks are 58-85% effective | (22) |

# SI References

1. Q. Lin *et al.*, A conceptual model for the outbreak of Coronavirus disease 2019 (COVID-19) in Wuhan, China with individual reaction and governmental action. *International journal of infectious diseases* (2020).

2. L. Peng, W. Yang, D. Zhang, C. Zhuge, L. Hong, Epidemic analysis of COVID-19 in China by dynamical modeling. *arXiv preprint arXiv:2002.06563* (2020).

3. J. M. Read, J. R. Bridgen, D. A. Cummings, A. Ho, C. P. Jewell, Novel coronavirus 2019-nCoV: early estimation of epidemiological parameters and epidemic predictions. *MedRxiv* (2020).

4. L. Y. Sanche S, Xu C, Romero-Severson E, Hengartner N, Ke R., High contagiousness and rapid spread of severe acute respiratory syndrome coronavirus 2. *Emerg Infect Dis.* **26** (2020).

5. J. Jia *et al.*, The impact of multilateral imported cases of COVID-19 on the epidemic control in China. *arXiv preprint arXiv:2004.02398* (2020).

6. Y. Dong *et al.*, Epidemiology of COVID-19 among children in China. *Pediatrics* (2020).

7. A. Kimball, Asymptomatic and presymptomatic SARS-CoV-2 infections in residents of a long-term care skilled nursing facility—King County, Washington, March 2020. *MMWR. Morbidity and mortality weekly report* **69** (2020).

8. H. Qiu *et al.*, Clinical and epidemiological features of 36 children with coronavirus disease 2019 (COVID-19) in Zhejiang, China: an observational cohort study. *The Lancet Infectious Diseases* (2020).

9. B. F. Maier, D. Brockmann, Effective containment explains subexponential growth in recent confirmed COVID-19 cases in China. *Science* **368**, 742-746 (2020).

10. C. M. Peak *et al.*, Modeling the comparative impact of individual quarantine vs. active monitoring of contacts for the mitigation of COVID-19. *medRxiv* (2020).

11. K. Prem *et al.*, The effect of control strategies to reduce social mixing on outcomes of the COVID-19 epidemic in Wuhan, China: a modelling study. *Lancet Public Health* **5**, e261-e270 (2020).

12. J. A. Backer, D. Klinkenberg, J. Wallinga, Incubation period of 2019 novel coronavirus (2019-nCoV) infections among travellers from Wuhan, China, 20–28 January 2020. *Eurosurveillance* **25**, 2000062 (2020).

13. W.-j. Guan *et al.*, Clinical characteristics of coronavirus disease 2019 in China. *New England journal of medicine* **382**, 1708-1720 (2020).

14. Q. Li *et al.*, Early transmission dynamics in Wuhan, China, of novel coronavirus–infected pneumonia. *New England Journal of Medicine* (2020).

15. D. Wang *et al.*, Clinical characteristics of 138 hospitalized patients with 2019 novel coronavirus–infected pneumonia in Wuhan, China. *Jama* **323**, 1061-1069 (2020).

16. F. Zhou *et al.*, Clinical course and risk factors for mortality of adult inpatients with COVID-19 in Wuhan, China: a retrospective cohort study. *The lancet* (2020).

17. W. C. Roda, M. B. Varughese, D. Han, M. Y. Li, Why is it difficult to accurately predict the COVID-19 epidemic? *Infectious Disease Modelling* (2020).

18. C. COVID, R. Team, Severe outcomes among patients with coronavirus disease 2019 (COVID-19)—United States, February 12–March 16, 2020. *MMWR Morb Mortal Wkly Rep* **69**, 343-346 (2020).

19. R. Verity *et al.*, Estimates of the severity of COVID-19 disease. *MedRxiv* (2020).

20. C. Wu *et al.*, Risk factors associated with acute respiratory distress syndrome and death in patients with coronavirus disease 2019 pneumonia in Wuhan, China. *JAMA internal medicine* (2020).

21. X. Yang *et al.*, Clinical course and outcomes of critically ill patients with SARS-CoV-2 pneumonia in Wuhan, China: a single-centered, retrospective, observational study. *The Lancet Respiratory Medicine* (2020).

22. N. C. Brienen, A. Timen, J. Wallinga, J. E. Van Steenbergen, P. F. Teunis, The effect of mask use on the spread of influenza during a pandemic. *Risk Analysis: An International Journal* **30**, 1210-1218 (2010).
